# Adapting to a Warmer Ocean—Seasonal Shift of Baleen Whale Movements over Three Decades

**DOI:** 10.1371/journal.pone.0121374

**Published:** 2015-03-18

**Authors:** Christian Ramp, Julien Delarue, Per J. Palsbøll, Richard Sears, Philip S. Hammond

**Affiliations:** 1 Mingan Island Cetacean Study, St. Lambert, Quebec, Canada; 2 Sea Mammal Research Unit, Scottish Oceans Institute, University of St. Andrews, St. Andrews, United Kingdom; 3 Marine Evolution and Conservation, Centre for Ecological and Evolutionary Studies, University of Groningen, Groningen, The Netherlands; UC Santa Cruz Department of Ecology and Evolutionary Biology, UNITED STATES

## Abstract

Global warming poses particular challenges to migratory species, which face changes to the multiple environments occupied during migration. For many species, the timing of migration between summer and winter grounds and also within-season movements are crucial to maximise exploitation of temporarily abundant prey resources in feeding areas, themselves adapting to the warming planet. We investigated the temporal variation in the occurrence of fin (*Balaenoptera physalus*) and humpback whales (*Megaptera novaeangliae*) in a North Atlantic summer feeding ground, the Gulf of St. Lawrence (Canada), from 1984 to 2010 using a long-term study of individually identifiable animals. These two sympatric species both shifted their date of arrival at a previously undocumented rate of more than 1day per year earlier over the study period thus maintaining the approximate 2-week difference in arrival of the two species and enabling the maintenance of temporal niche separation. However, the departure date of both species also shifted earlier but at different rates resulting in increasing temporal overlap over the study period indicating that this separation may be starting to erode. Our analysis revealed that the trend in arrival was strongly related to earlier ice break-up and rising sea surface temperature, likely triggering earlier primary production. The observed changes in phenology in response to ocean warming are a remarkable example of phenotypic plasticity and may partly explain how baleen whales were able to survive a number of changes in climate over the last several million years. However, it is questionable whether the observed rate of change in timing can be maintained. Substantial modification to the distribution or annual life cycle of these species might be required to keep up with the ongoing warming of the oceans.

## Introduction

Migration occurs in all major branches of the animal kingdom, takes place in the air, at sea and on land, and exists at extreme temporal and spatial scales[1]. It can take many forms ranging from diurnal vertical migration of plankton to the migration of diadromous fish species to and from their spawning sites [2,3]. The main driving force is typically the use of resources such as food, mates or shelter varying over time and space [4].We focus here on the “classic” annually reoccurring seasonal large-scale migration between summer and winter areas [1,5]. Such migratory species may be subject to climate-induced changes in resources at different seasonal and life cycle stages to which they must adapt, or adjust their migratory behaviour [6]. The timing of these changes may differ in direction, severity and speed [7], potentially resulting in a temporal mismatch between resources and migratory cycles [8].

Seasonally migrating species have been shown to change their home ranges in both summer and wintering areas [9,10] or alter their timing in response to changes in their environment [11]. Numerous migratory species utilize high-latitude summer regions to benefit from the temporarily available high productivity and some of them reproduce during that period [6,12,13]. The timing of arrival at such summer foraging areas often corresponds with the occurrence of one or several prey species on which the adults or their offspring depend [8]. Increasing spring temperatures have shifted the phenology of plants and insects so that species at higher trophic levels have modified their timing patterns accordingly [14]. However, some avian species have failed to change their arrival timing sufficiently to maintain synchrony with their prey species [15], and similar trophic mismatches have been observed in the marine ecosystem [16]. While most migratory species show a unidirectional reaction to warmer temperatures in spring, the effect on autumn departure varies. Short-distance migrants tend to depart later in autumn whereas long-distance migrants depart earlier [11,17]. Generally, long-distant migrants seem to adapt less well to climate change than short-distant migrants potentially leading to reduced fitness and population declines. Animals cannot predict environmental conditions thousands of kilometers away and potential wrong timing of movements causing a mismatch with prey occurrence might reduce feeding success, and thus reproductive success and ultimately survival [17,18].

Most baleen whales undertake seasonal migrations ranging from a few hundreds to thousands of kilometers [19–21] alternating between low latitude winter breeding grounds and high latitude summer feeding grounds [22,23]. Baleen whales have existed for several million years [24] and have thus survived several glacial and interglacial periods, including the Dansgaard-Oeschger cycles, when temperature over Greenland increased 8–15°C in the span of a few decades on multiple occasions during the last 80,000 years [25,26]. These species have lifespans of at least 40 to well over 100 years [27,28] and individual whales thus experience more environmental variation during their life-time than individuals of most other species. The large body size of baleen whales helps buffer individuals against short-term variation in environmental conditions and reduces the relative costs of locomotion [29,30], facilitating long-range seasonal migration and extensive movements across the summer range [31,32].

With such advantages, how are baleen whales adapting to global warming? Migratory species have been described as a ‘paradox’ because their mobility allows them to react even to rapid changes but they also depend on suitable habitat in multiple locations [6]. Predictions for the response of marine mammals range from a more pole-ward distribution and the earlier arrival in feeding areas to follow changing prey distribution [33,34] to a longer residency time of some migratory species in higher latitudes in response to enhanced productivity [33]. Several studies suggest that entire ecosystems or communities will move pole-ward following rising SST [35,36].

Populations of fin (*Balaenoptera physalus*) and humpback whales (*Megaptera novaeangliae*), the target species of this study, spend part of the summer in the Gulf of St. Lawrence in the North Atlantic Ocean. These two species are sympatric and feed on a wide, mostly overlapping, variety of zooplankton and schooling fish [37]. They exhibit some niche separation in the Gulf of St. Lawrence; fin whales arrive earlier and feed, on average, at a lower trophic level and wider niche than humpback whales, which arrive later and feed on higher trophic prey that are more available later in the season [38,39]. Northwest Atlantic humpback whales breed in the West Indies during winter [40] after traveling between 2,000 and 8,000 kilometers from their summer feeding grounds, which include the Gulf of Maine, eastern Canada, and western Greenland [41,42]. The fin whales found in the summer in the Gulf of St. Lawrence are believed to overwinter off Nova Scotia just outside the pack ice [43], but their winter distribution is poorly known [44]. Indeed, most of their entire range and migration routes are unknown and they are thought to be more pelagic because they are not observed as close to the shore as are humpback whales and do not seem to aggregate in distinct breeding grounds.

The best documented effects of recent changes in climate are the warming of the oceans and the reduction in sea ice [45]. The Gulf of St. Lawrence has a subarctic climate with seasonal ice cover in winter and spring, representing the southernmost extent of sea ice in the Northern Hemisphere [46]. Over the last 30 years, sea surface temperature (SST) in the Gulf of St. Lawrence has increased [47] while temporal ice coverage has decreased [48]. We investigated whether, and in which direction, fin and humpback whales have changed their temporal occurrence in this area in response to the warming climate. We used the timing of the first and last sighting of photographically identified individuals as proxies for the arrival and departure dates of these two species in the main feeding area in the Gulf of St. Lawrence over a period of 27 years. Many individuals were present in the area throughout the study period. Therefore, we not only use these individuals as samples to explore the potential response to climate change of the two populations, but also investigate the behavioural change or adaption of individuals.

We also investigate whether changes in the timing of occurrence are linked to changing environmental conditions in the area and, using this case study as a proxy for a feeding ground in general, explore possible future responses and implications for these species, faced with predicted continuing ocean warming. Our analysis reveals that the earlier arrival of fin and humpback whales in the Gulf of St. Lawrence by approximately one day per year over 27 years was strongly related to an earlier ice break-up and rising sea surface temperature, indicating a remarkable phenotypic plasticity to a changing environment.

## Material and Methods

### Data

**Fig 1:**
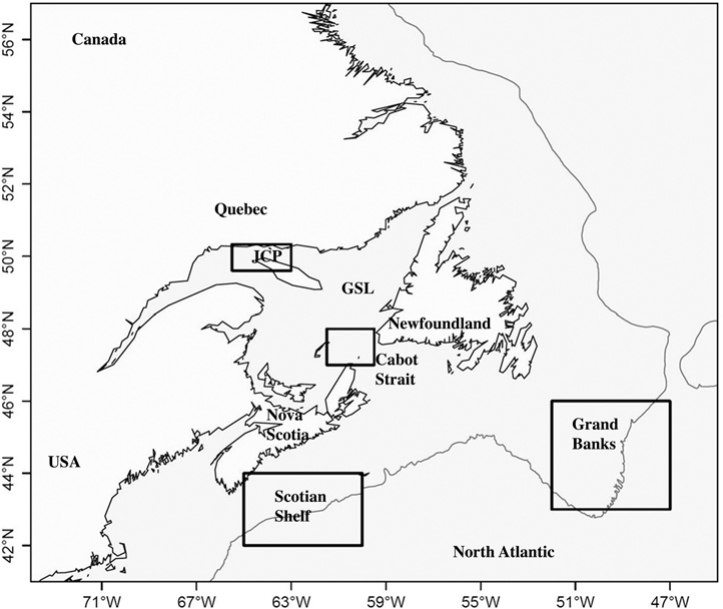
The Study area. The Gulf of St. Lawrence and eastern Canadian waters with 500m bathymetric line representing the shelf edge. The research area is marked as JCP (Jacques Cartier Passage). The other boxes show the extent of the areas for which SST data were used.

The Gulf of St. Lawrence is a semi-enclosed sea that connects the Great Lakes to the North Atlantic and is a summer feeding destination for several species of rorqual whales [49]. The data were collected in the Jacques-Cartier Passage and adjacent waters (roughly 49.5°N to 50.3°N and 63°W to 66°W (Fig. 1)) between 1984 and 2010. A field season lasted between the beginning of June and mid/end of October with an average of 65 survey days and ~500 hours of observation.

We used standard photo-identification techniques to identify individual humpback and fin whales from their natural markings [50,51]. The primary aim of the study was the estimation of population parameters using mark-recapture techniques and thus we maximised the effort to photo-identify all animals present. The sighting effort remained relatively stable over the study period. The starting date of the seasonal fieldwork remained relatively constant (S1 Table), while the end date depended on the animals; surveying stopped when no animals had been sighted in the study area for approximately two weeks. The study was conducted under annual permits from Department of Fisheries and Oceans, Canada.

Between 1984–2010, we identified 450 fin whales, which were sighted on a total of 1,404 occasions, and 270 individual humpback whales sighted 1,075 times in total. Not all juvenile humpback whales migrate to the breeding grounds [52] and might remain closer to the feeding grounds during winter. Therefore, we restricted our analysis to mature animals under the assumption that mature animals consistently migrate to the breeding grounds each year. Accordingly, we included only sightings of animals when they were known for at least 5 years, the age at which humpback whales are presumed to be sexually mature [53]. This reduced data set contained 96 individual humpback whales sighted 677 times in total. For fin whales, it is unknown whether or not all animals migrate but we assumed they do and used all sighting data in our analysis. However, calves (lactating young of the year) of both species were omitted because their occurrence depends on their mothers.

### Analysis

We used the first and last date in each year (1984–2010) that an individual whale was sighted and identified as proxies for its arrival and departure date, respectively, and the mean first and last sighting dates as estimates of the population mean arrival and departure date, respectively. Individual whales were not necessarily detected when present and true arrival and departure dates of each individual whale in each year were unknown. Mean date of first sighting is thus a potentially biased (late) estimate of population arrival date, mean last sighting date is a potentially biased (early) estimate of population departure date and residency times are thus potentially underestimated. This bias might be reduced by limiting analysis to data for animals seen multiple times, for example by removing animals seen only once in a year and thus with identical arrival and departure dates. We conducted analyses with datasets reduced in this way and obtained similar results to the simple regressions (S2 Table). We therefore used all data to maximise sample size and to avoid the risk of introducing unknown bias by setting a threshold for the amount of data available for individuals.

Mean first and last sighting dates were each regressed on year to estimate the annual rates of change. These dates for the same individual in different years could potentially be viewed as repeated measures, thus violating assumptions of independence when estimating trends over time. To test for bias, we randomly selected one observation per individual from the total dataset, calculated the annual mean first sighting day for that reduced dataset, regressed mean first sighting date against year, and repeated this procedure 1,000 times. We then compared the mean slope and 95% confidence interval (CI) of the reduced resampled data set with the regression estimated using the full dataset. The difference in slope between the full and the reduced resampled data sets was negligible and the slopes of the linear models using all data fell within the 95% confidence interval of the replicated data sets (S1 and S2 Figs.). Thus we based our analyses on the full data set. We repeated this bootstrap procedure for the last sighting (departure) date (S3 and S4 Figs.), which gave similar results. As an alternative way of addressing this issue, we also fitted mixed effects models, which gave very similar results to the simple regressions (S2 Table).

We subsequently developed a set of linear regression models with annual mean first sighting date as the response variable to investigate the explanatory power of a range of covariates (Table 1) [54,55]. The covariates included several measures of research effort to test that the trend was not influenced by effort (S1 Table). The environmental covariates included the large-scale North Atlantic Oscillation (NAO) index, sea surface temperatures (SST), and measures of ice coverage in the Gulf of St. Lawrence. Timing of arrival of whales in the St. Lawrence is assumed to depend on the availability of prey. Prey data were unavailable for our research area and chlorophyll concentration was available only for the later years of the study so we used the ice and SST covariates as a proxy for the onset of annual primary productivity [46].

**Table 1:** Covariates used in the linear regression modeling.

Covariate	Data period	Comment
**Effort:**		
First survey day of the year	1985–2009	
Number of survey days in June	1985–2009	
Number of survey days in July	1985–2009	
Number of survey days in June+July	1985–2009	
**Sea ice data**		
First week ice free	1985–2009	< 1% ice coverage
No. of weeks with ice	1985–2009	> 1% ice coverage
Week number with maximum ice coverage	1985–2009	
**Climate data**		
NAO monthly anomalies	1985–2009	9 monthly means prior arrival
Mean and Maximum monthly SST	1985–2009	
SST in GSL6 [55]	1985–2009	9 monthly means prior arrival
SST in Cabot Strait (CB) (Fig. 1)	1985–2009	9 monthly means prior arrival
SST in Grand Banks (GB) (Fig. 1)	1985–2009	9 monthly means prior arrival
SST in Scotian Shelf (Fig. 1)	1985–2009	9 monthly means prior arrival

Sea ice data were obtained from Environment Canada Ice Services (http://ice-glaces.ec.gc.ca/). We obtained mean and maximum monthly Sea Surface Temperatures (SST) using AVHRR Pathfinder Version 5.2 (PFV5.2) data, obtained from the US National Oceanographic Data Center and GHRSST (http://pathfinder.nodc.noaa.gov). The PFV5.2 data are an updated version of the Pathfinder Version 5.0 and 5.1 collection described in [54]. The North Atlantic Oscillation (NAO) monthly anomalies were downloaded directed from NOAA:ftp://ftp.cpc.ncep.noaa.gov/wd52dg/data/indices/tele_index.nh. GSL6 is a region along the Quebec North Shore, covering our study area [55]. SST was only available from 1985 to 2009 and therefore we used the same time frame for all other variables.

We also wanted to test the hypothesis that time of arrival is also influenced by the conditions of the area the animals are leaving. The winter and spring distribution of fin whales is not well known, but some reports suggest that they spend the winter just outside the Gulf of St. Lawrence [43] and large whales are reported on Grand Banks in spring [56]. Therefore, we also included as candidate covariates SST data from several regions outside the Gulf (Fig. 1 and Table 1). There are time lags of several weeks between the ice break up, rising temperatures, start and peak of primary production and the occurrence of prey species. Therefore we investigated monthly means of SST of 1 to 9 months prior to the earliest arrival (October to June). SST data were not available for 1984 and 2010. Thus, we restricted this analysis to 1985 to 2009, for which data were available for all covariates.

We used simple least squares regression and examined the variance explained in models for each covariate alone. Monthly mean SST is not independent within regions and seasons, nor likely to be between regions, so we included only a single SST variable in each model. Similarly, ice breakup, duration and coverage are also highly correlated and we used only a single ice-related covariate in each model. Although ice breakup, coverage and duration are also correlated with sea surface temperature and all of these are correlated with climate variables such as the NAO index, we decided to test two variables at a time and to choose which of the SST and ice-related variables to include based on significance tests and variance explained by the models.

Model selection was based on Akaike’s Information Criterion [57]. All statistical analysis was undertaken in R [58].

## Results

**Fig 2:**
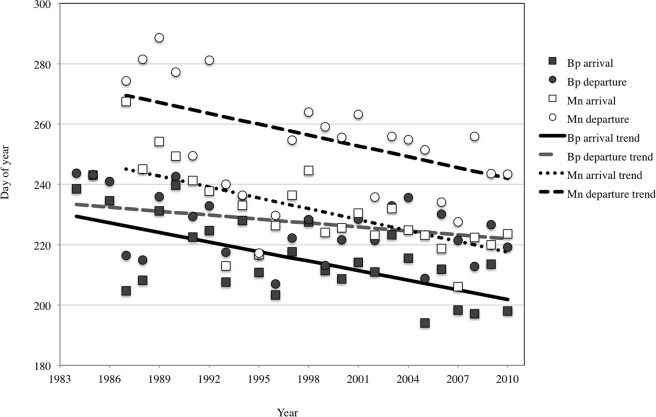
Mean arrival and departure date of fin (Bp) and humpback whales (Mn). The difference between trends in arrival and departure represent average measured residency time.

The mean first sighting date of fin whales shifted significantly earlier over the study period (slope = -1.062, SE = 0.093, p<0.001), from day 229 (17 August) in 1984 to day 201 (20 July) in 2010 (Fig. 2), thus one month earlier over 27 years. The mean first sighting date of mature humpback whales (Fig. 2) also shifted significantly earlier (slope = -1.201, SE = 0.135, p <0.001); from day 245 (2 September) in 1987, to day 217 (5 August) in 2010 (Fig. 2). Plots of the standardized residuals of the models showed no departure from normality nor evidence for temporal correlation (S5–S8 Figs.).

The mean last sighting date of fin whales also moved earlier in time (slope = -0.435, SE = 0.09, p<0.001) from day 233 (21 August) to day 222 (10 August) but the change was not as great as for first sighting date (Fig. 2). Thus fin whales extended the period between first and last sighting date by an average of 16 days across the study period. The mean day of the last sighting for mature humpback whales also changed over the study period (slope = -1.196, SE = 0.136, p<0.001) from day 269 (26 September) to day 242 (30 August), resulting in a constant average period between first and last sighting during the 27-year study period.

**Fig 3:**
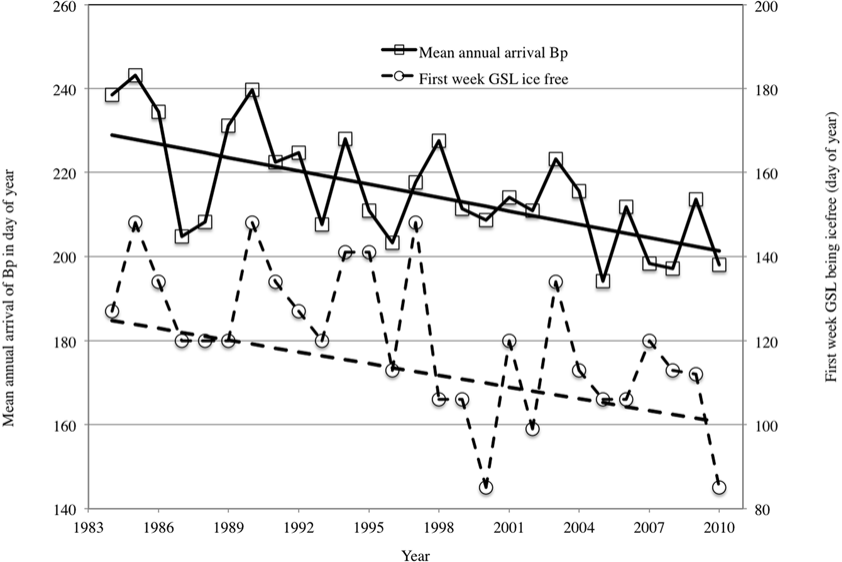
Mean annual arrival date of fin whales (Bp) and the first week the Gulf of St. Lawrence was ice-free.

In the regression models for fin whales (Table 2) many covariates were significant but they accounted for only a small fraction of the variance in date of first sighting. No effort variable was significant. We tested multiple combinations of significant covariates but only two improved the model fit. The best-supported model (adjusted r^2^ = 0.51) included the first ice-free week in the Gulf of St Lawrence (slope = 0.32, SE = 0.11, p = 0.013, Fig. 3) and sea surface temperature (SST) in Cabot Strait in January (slope = -8.5, SE = 2.6, p = 0.003). NAO was significant as a single variable but not in combination with any other covariate. For mature humpback whales, many of the covariates were also significant in the models and explained some variation in the first sighting date (Table 3). No effort variable was significant. The best-supported model (adjusted *r* ^2^ = 0.47) included a single covariate, the SST at the research site in January (slope = -33.66, SE = 7.45, p = 0.001). No combination of additional covariates improved the overall model fit.

**Table 2:** Model results Fin whales.

model	intercept	slope	r^2^	AICc	ΔAICc	AICc weight
ice free + SST.CB.JAN	175.9	0.328 / -8.52	0.513	187.9	0	0.792
SST.CB.JAN	215.8	-10.97	0.377	192.3	4.39	0.088
SST.GS6L.OCT	259.4	-6.705	0.345	193.6	5.66	0.047
ice free + SST.GSL6.OCT	214.3	0.248 / -4.38	0.372	194.3	6.35	0.033
ice free	159.7	0.464	0.31	194.9	6.96	0.024
No weeks ice	167.3	2.502	0.22	197.7	9.77	0.006
SST.GSL6.JAN	192.7	-19.79	0.202	198.5	10.54	0.004
NAO March	213.1	6.246	0.173	199.4	11.5	0.003
Week max ice	250.4	-0.577	0.166	199.6	11.71	0.002
Days effort July	236	-1.106	ns	203.5	15.53	0
Total effort J/J	225.9	-0.358	ns	204.3	16.38	0
First day effort	176	0.245	ns	204.4	16.46	0
Days effort July	218.7	-0.271	ns	205	17.04	0

Selection of models for fin whale arrival (1985–2009) ordered by AICc. r^2^ values given only for models in which the covariates was significant. ns stands for non-significant covariates

**Table 3:** Model Results Humpback whales.

model	intercept	slope	r^2^	AICc	ΔAICc	AICc weight
SST.GSL6.JAN	192.6	-33.67	0.47	178.3	0	0.76
SST.GB.JAN	253.5	-6.50	0.27	180.9	2.62	0.205
SST.CB.JAN	231.7	-9.94	0.2	185.7	7.46	0.018
NAO.OCT	234.4	6.51	0.15	187.8	9.54	0.006
SST.GSL.DEC	238	-10.01	0.09	189.1	10.86	0.003
First day effort	336.1	-0.65	ns	190.6	12.37	0.002
SST.CB.DEC	243.2	-4.50	ns	191.2	12.92	0.001
No weeks ice	197.4	1.74	ns	191.4	13.18	0.001
First week ice free	200.9	0.25	ns	191.5	13.29	0.001
Days effort June	223.1	0.78	ns	192.2	13.99	0.001
Week max ice	216.8	-0.20	ns	192.8	14.57	0.001
Total effort J/J	243.3	0.05	ns	193.3	15.01	0
Days effort July	230.4	0.04	ns	193.6	15.33	0

Selection of models for mature humpback whale arrival ordered by AICc. r^2^ values given only for models in which the covariate was significant. ns stands for non-significant covariates. Only the covariates of the first five models were significant. No combination with two covariates yielded significant results.

## Discussion

Inferring from the results for mean first sighting date, fin and humpback whales arrived earlier in the study area over the 27 years of the study, in line with general predictions [34]. However, the rate of change of >1day earlier per year is, to our knowledge, undocumented. Both species also left the study area earlier, as inferred from mean last sighting date, as observed in many other species [11,17]. Humpback whale departure shifted at the same pace as arrival, thus keeping the residency time almost constant. The trend towards an earlier departure date of fin whales was less pronounced than for the arrival date, increasing the residency time, but that increase is subject to small sample bias in the first two years. Thus, there is only weak evidence that fin whales increased their residency time in the study area, and none for humpback whales.

The Jacques Cartier Passage is an important summer feeding area for both species, with many individuals returning every year, as indicated by high recapture rates [59,60], but it represents only a fraction of the potential summer range for both populations. Individuals of both species thus likely spent only a part of the summer in our study area and the residence times calculated here do not represent their entire feeding season. Fin whales are thought to spend the winter outside the Gulf of St. Lawrence and move in with the retreating ice [43] and then follow the plankton bloom further north [61], but their winter distribution is unknown. For humpback whales, we know when they depart from the breeding grounds [62] and it is clear that the arrival date calculated here does not represent the arrival direct from the wintering grounds. They arrive in the feeding latitudes already in May and June and stay until October or even later [62]. However, whether we consider the classic migration from the winter grounds or the within season movements between different summer feeding areas, both species showed the same behavioural adaptation and advanced their temporal occurrence in the Jacques Cartier Passage by one month.

The observed change in phenology of these populations is based on the observation of many re-sighted whales over the study period, showing remarkable adaptation of the individual animals to changing conditions and highlighting the phenotypic plasticity of these species. We observed many of the same individuals at the beginning and the end of the study period, thus there is no evidence that animals left the study area and moved pole-ward, as some studies have predicted [33]. However, our results are limited by the size of the study area and animals could have probed further north before or after their occurrence in the study area.

Fin whale arrival in the Gulf of St. Lawrence followed the shift in the date of the ice break up, as first suggested many years ago [43]. The influence of SST inside Cabot Strait (Fig. 1) is likely related to the spatial and temporal ice coverage and could serve as a signal to the whales that it is time to move back into the Gulf of St. Lawrence. There was a time lag of 13–15 weeks between when this area became totally ice-free and the arrival of the fin whales in the Jacques Cartier Passage. This period is similar to the time lag estimated in the Azores, where fin and humpback whales appear to arrive 15 weeks after the onset of the spring bloom to feed on euphausiid species when *en route* to high latitude summer feeding grounds [61]. For humpback whales, we assume that the influence of SST in January in the Gulf of St. Lawrence on arrival date must constitute a proxy for larger scale environmental variation because at this time humpback whales are in the West Indies on the breeding grounds approximately 4,000 kilometers south of the Gulf of St. Lawrence. However, the only large-scale climate variable included in our models, the North Atlantic Oscillation index, did not correlate well with the observed change in arrival date. It is unknown where humpback whales are located between their arrival in higher latitudes from the breeding grounds and their arrival in the Jacques Cartier Passage but environmental changes may have triggered an earlier departure from this unknown location and thus earlier arrival in the Gulf of St. Lawrence.

Fin and humpback whales are generalist feeders and the arrival of the whales in the Gulf of St Lawrence is related to the temporal and spatial distribution of the arrival of their prey. The start of the spring phytoplankton bloom depends on temperature and light conditions [16] and earlier ice break-up coupled with higher SST leads to a progressively earlier bloom followed by the earlier growth of populations of primary and then secondary consumers. Thus, the earlier arrival of fin and humpback whales enables timely feeding on these prey species.

Humpback whales arrive in the Gulf of St Lawrence about 2 weeks later than fin whales. Analysis of the isotopic niche in this area between 1992 and 2010 revealed that humpback whales fed at a higher trophic level compared to fin whales [38] implying that the difference in timing of arrival may reflect a difference in prey preference. A possible explanation for the observed temporal separation between fin and humpback whales is niche partitioning to reduce competition. Our results show that the temporal separation between these two species has so far largely been maintained despite the shift towards earlier arrival times. However, our results also show that this temporal separation may have started to erode due to the weak evidence for longer residency times of fin whales.

Short-distance migrants seem to adapt better to climate change than long-distant migrants [17,18]. Some Northern Hemisphere bird species have shortened their migration distance by establishing wintering grounds further north [10,63], while other bird species have ceased to migrate altogether [64]. For baleen whales, gray whale (*Eschrichtius robustus*) calls have been recorded in the winter off Alaska, indicating that some individuals may have ceased to migrate annually [65]. While bird species breed and feed at high latitudes during the summer, most baleen whales feed only during the summer. When sea ice covers the Gulf of St. Lawrence, fin whales are assumed to winter only a few hundred kilometers away off the coast of Nova Scotia [43]. As the winter sea ice coverage decreases in the Gulf of St. Lawrence, so may the need for fin whales to migrate. A subpopulation of fin whales in the Mediterranean Sea that is distinct from conspecifics in the North Atlantic does not migrate long distances but feeds during the winter and may reproduce year-round [66–68]. If the patterns described here for fin whales in the Gulf of St. Lawrence continue, and noting that they show flexibility regarding where they give birth [23], it is tempting to speculate that continuing warming could lead to a discrete year-round population of fin whales in the Gulf of St Lawrence if parts of it become ice-free in winter.

There is, to our knowledge, no indication that humpback whales have shifted their timing of arrival at or departure from their breeding grounds in the West Indies. The results presented here indicate, rather, an earlier inshore movement within northern latitudes. Humpback whale feeding aggregations throughout the North Atlantic show a wide variation in migration distances [41]. Those populations feeding at temperate latitudes may simply extend their annual migration further north as their prey retreat northbound in response to elevated SST [69], as several studies have predicted, thus increasing interspecific competition with northern species [70,71]. The continuing rise in ocean temperatures could eventually cause problems for long distance migrating humpback whales to time their arrival in the feeding grounds with the occurrence of their main prey. Extreme changes, such as shown in this study, and pressure to adapt further to accommodate ongoing rising SST are likely to affect population dynamics as shown for other marine migrants [72,73] and future studies should test for effects of climate change on population dynamics and health.

Fin and humpback whales in the Gulf of St. Lawrence have shifted their phenology at a previously undocumented pace over the last 27 years. Both species have adapted their seasonal movement to the shift in productivity in one of their prime feeding grounds in the North Atlantic. Whether this pattern can continue as ocean temperatures increase is an open question and the implications for these two species in the region are uncertain but could be profound. The phenotypic plasticity of these long-lived marine predators shown in our analysis is notable and may explain how they have coped with past fluctuations in climate. However, it remains questionable for how much longer they can adapt to further rapid environmental change. Substantial modification to their distribution or annual life cycle might be required to keep up with the continuing warming of the oceans, and the implications might be more severe for the humpback whale with more distant breeding grounds.

## Supporting Information

S1 Fig Arrival date of individual fin whales. All first sightings with the linear trend (red line, slope = -1.062, SE = 0.093) laying within the upper and lower 95% confidence intervals (dashed black lines) of the resampled data. Annual trend of resampled data as black line (bootstrapped data slope = -1.097, SE = 0.006).
(PDF)

S2 Fig Arrival date of individual (mature) humpback whales. All first sightings with the linear trend (red line, slope = -1.201, SE = 0.135) laying within the upper and lower 95% confidence intervals (dashed line) of the resampled data. Annual trend of resampled data as black line (bootstrapped data slope = -1.041, SE = 0.016).
(PDF)

S3 Fig Departure date of individual fin whales. All first sightings with the linear trend (red line) laying within the upper and lower 95% confidence intervals (dashed black lines) of the resampled data.
(PDF)

S4 Fig Departure date of individual humpback whales. All first sightings with the linear trend (red line) laying within the upper and lower 95% confidence intervals (dashed line) of the resampled data.
(PDF)

S5 Fig Standardized residuals over standardized predicted values of the linear model (arrival~year) for the fin whale data set. (PDF)

S6 Fig Histogram of standardized residuals of the linear model (arrival~year) with theoretical normal distribution of the fin whale data set. (PDF)

S7 Fig Standardized residuals over standardized predicted values of the linear model (arrival~year) for the mature humpback whale data set. (PDF)

S8 Fig Histogram of standardized residuals of the linear model (arrival~year) with theoretical normal distribution for the humpback whale data set. (PDF)

S1 File Raw data. Arrival and Departure Dates for all Individuals.
(PDF)

S1 Table Dataset. Mean arrival and departure date for fin and (mature) humpback whales and the effort covariates according to Table 1 (No of survey days in June, July, June and July, and the first survey day of the year)
(DOCX)

S2 Table Model Results. Estimated regression slopes (SE) for the annual trend in arrival, using different subsets of the data and different model approaches. Numbers in bold italics indicate results used in the manuscript. In mixed effect models, individual whales were defined as random effects.
(DOCX)
