# Supplementary figures and images for: Adapting to a Warmer Ocean—Seasonal Shift of Baleen Whale Movements over Three Decades
Source: PLoS One. 2015 Mar 18;10(3):e0121374. doi: 10.1371/journal.pone.0121374 (PMC4364899; doi:10.1371/journal.pone.0121374)

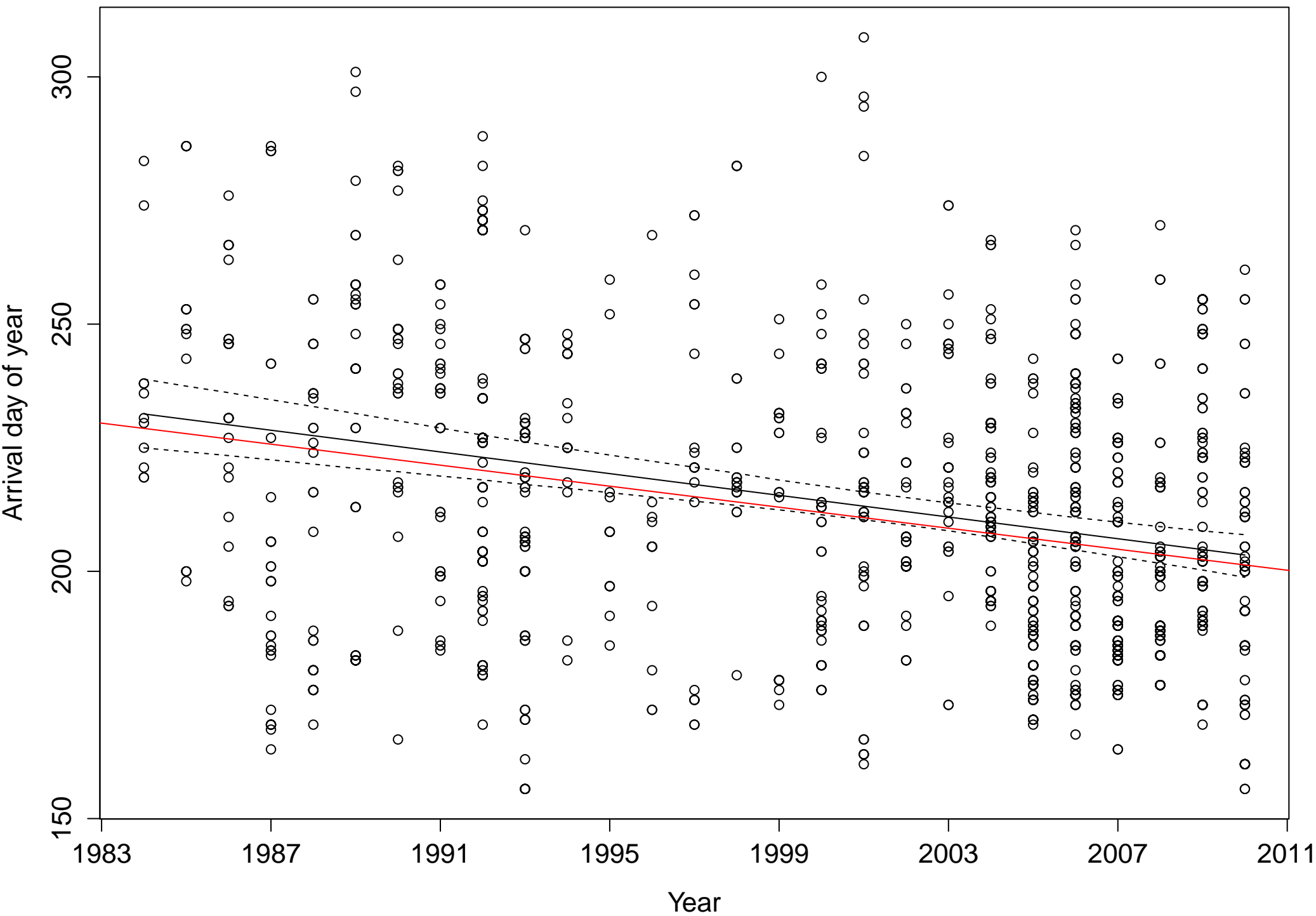

Supplement: S1 Fig — All first sightings with the linear trend (red line, slope = -1.062, SE = 0.093) laying within the upper and lower 95% confidence intervals (dashed black lines) of the resampled data. Annual trend of resampled data as black line (bootstrapped data slope = -1.097, SE = 0.006). (PDF) [file pone.0121374.s001.pdf]

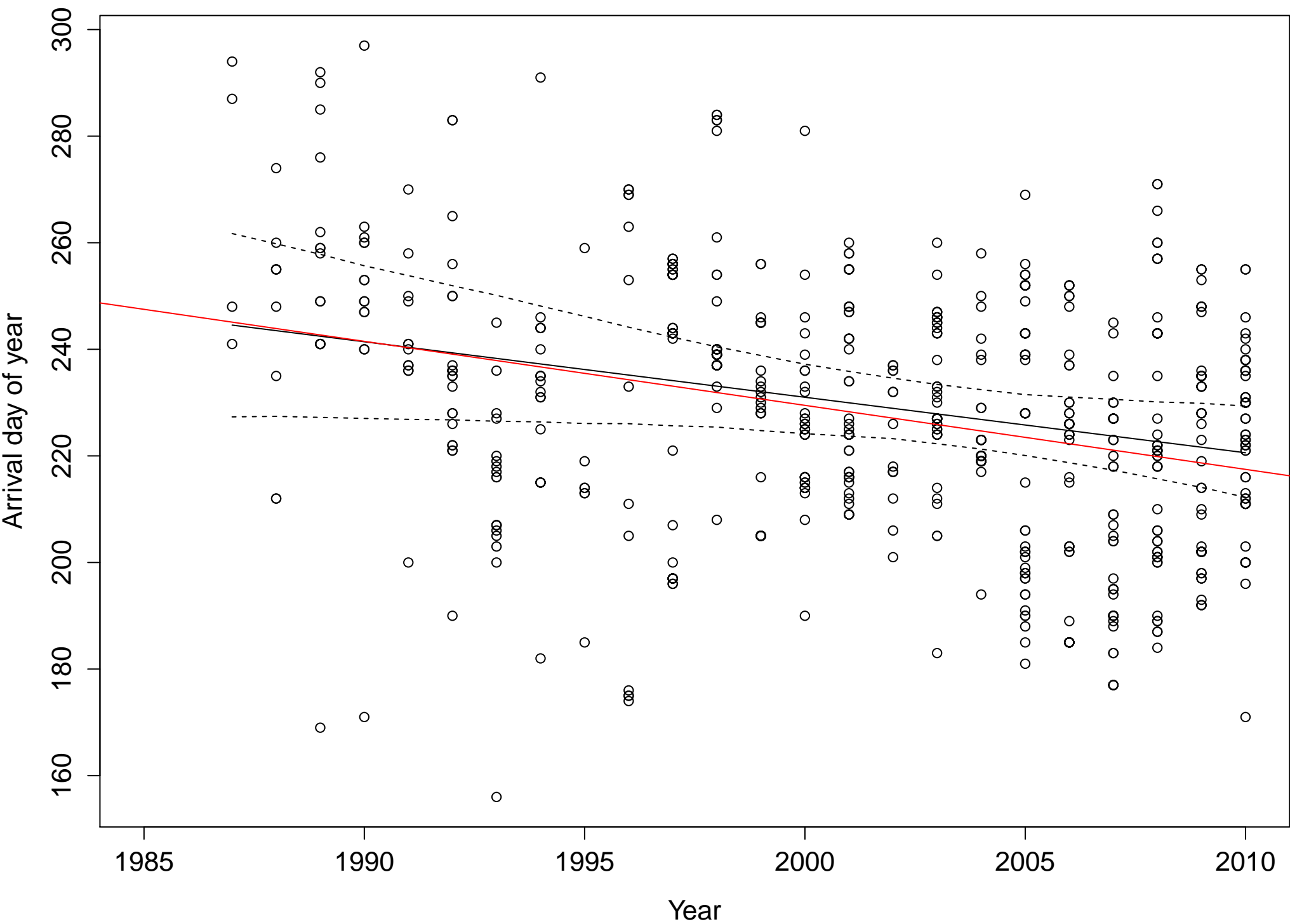

Supplement: S2 Fig — All first sightings with the linear trend (red line, slope = -1.201, SE = 0.135) laying within the upper and lower 95% confidence intervals (dashed line) of the resampled data. Annual trend of resampled data as black line (bootstrapped data slope = -1.041, SE = 0.016). (PDF) [file pone.0121374.s002.pdf]

Departure day of year

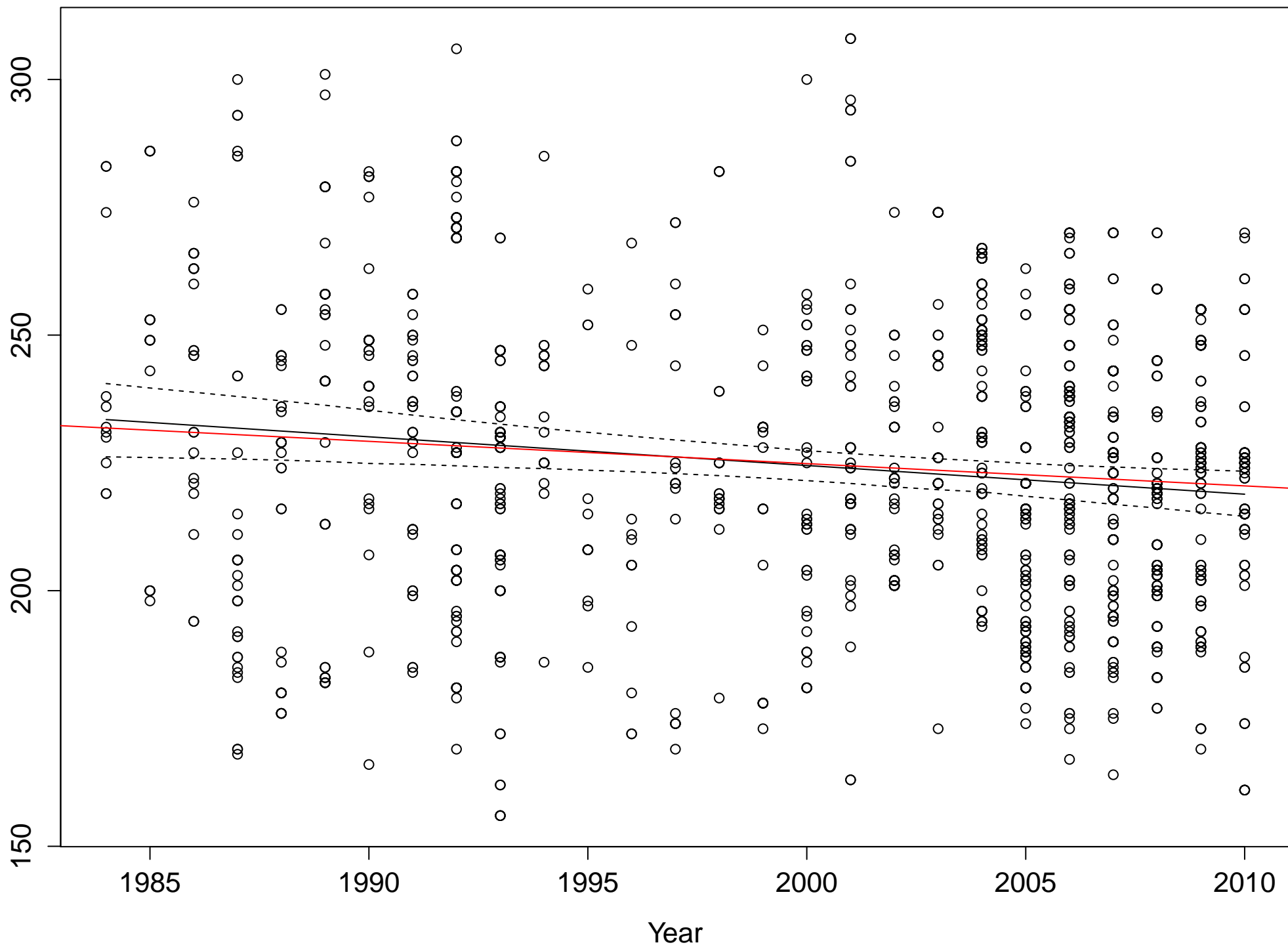

Supplement: S3 Fig — All first sightings with the linear trend (red line) laying within the upper and lower 95% confidence intervals (dashed black lines) of the resampled data. (PDF) [file pone.0121374.s003.pdf]

Departure day of year

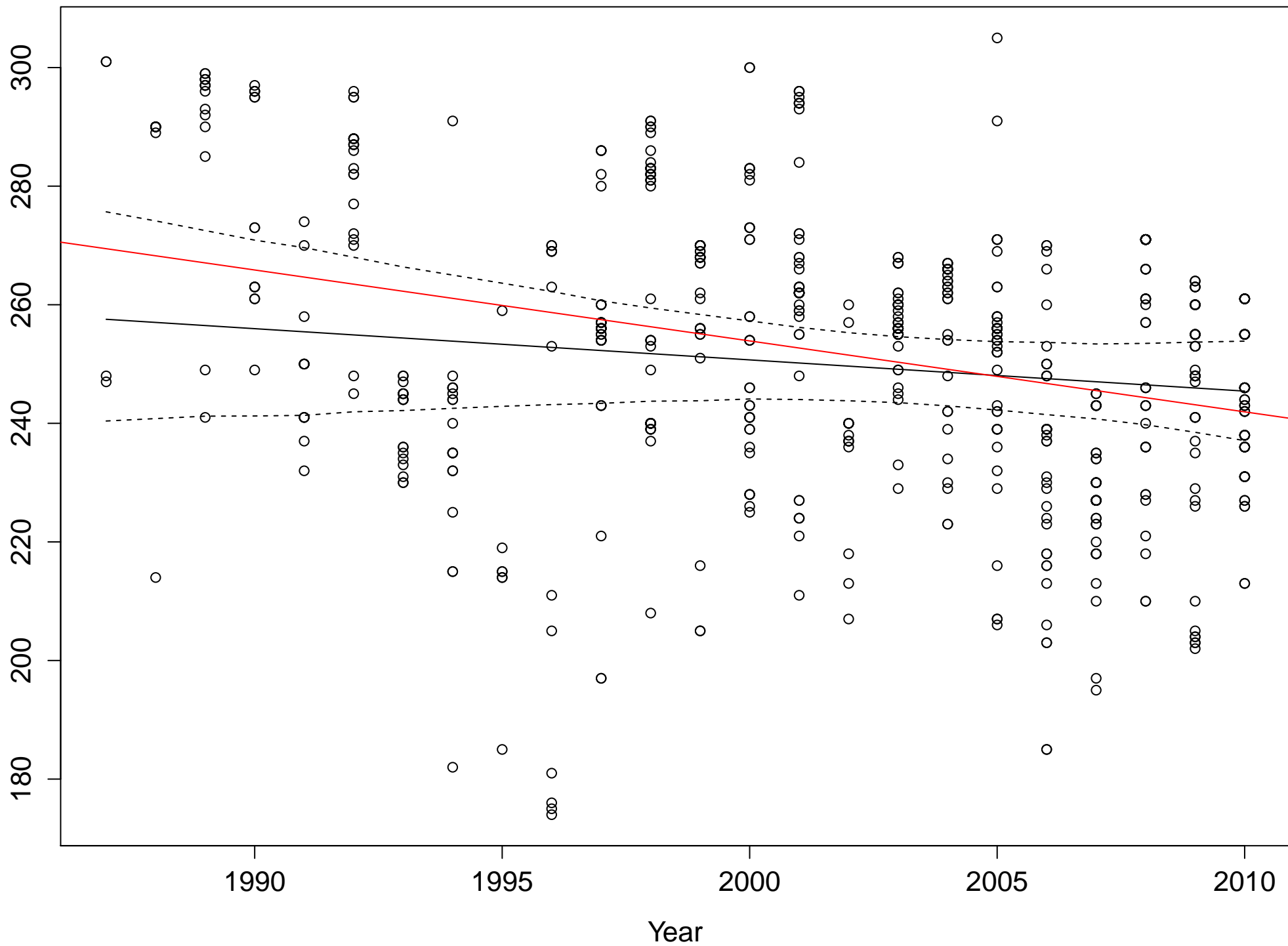

Supplement: S4 Fig — All first sightings with the linear trend (red line) laying within the upper and lower 95% confidence intervals (dashed line) of the resampled data. (PDF) [file pone.0121374.s004.pdf]

**Standardized Residuals Plot**

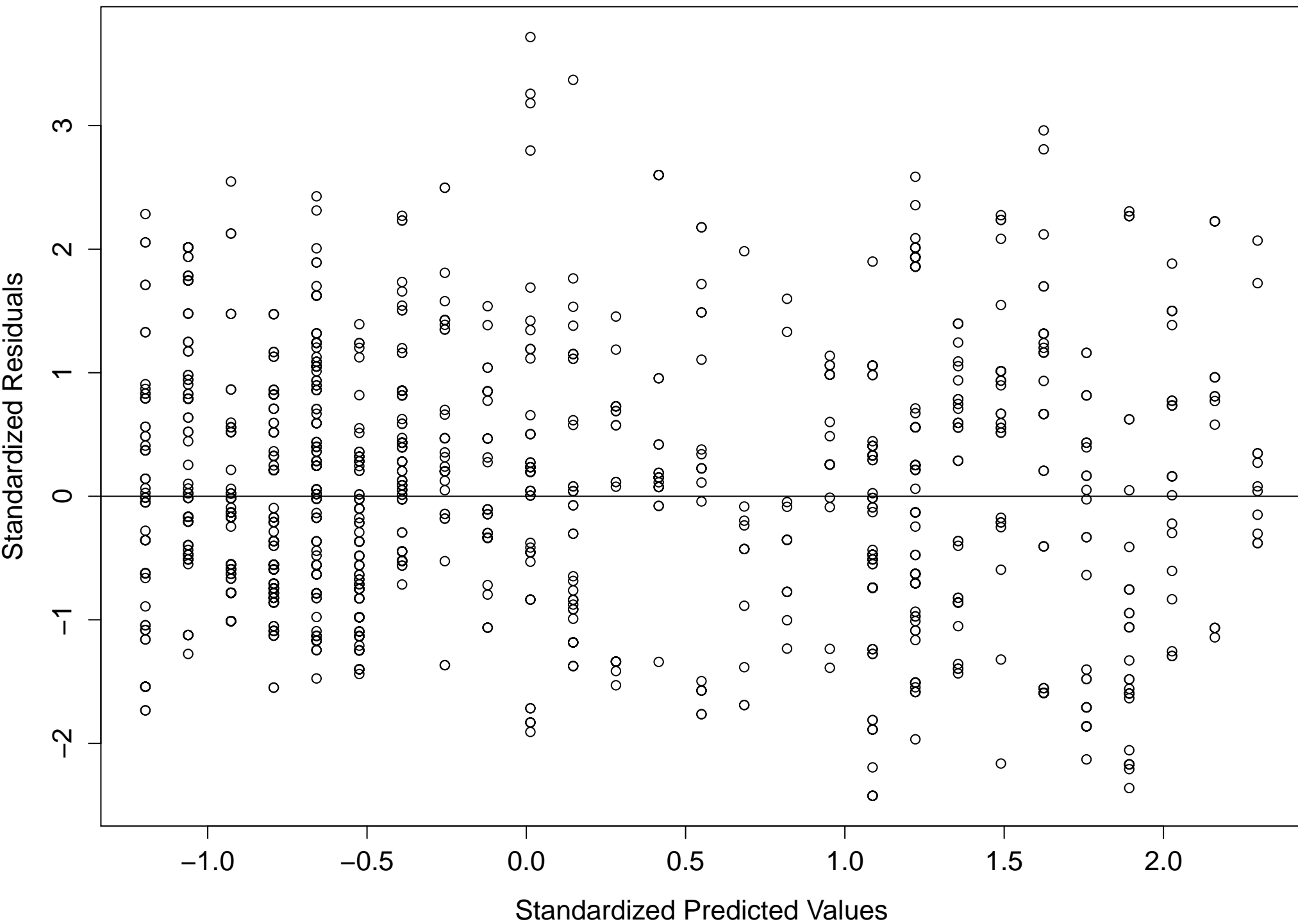

Supplement: S5 Fig — (PDF) [file pone.0121374.s005.pdf]

# Histogram of Standardized Residuals

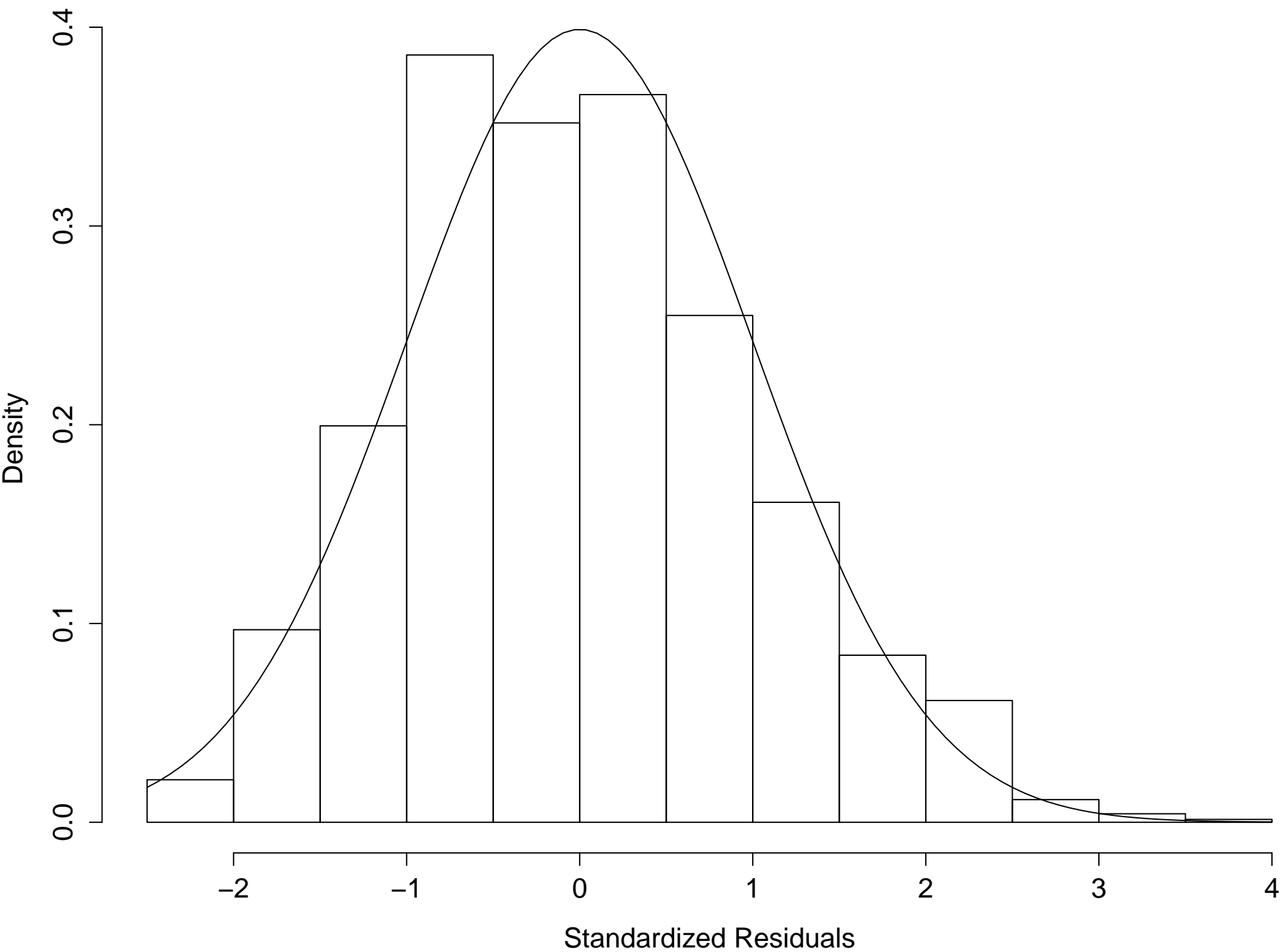

Supplement: S6 Fig — (PDF) [file pone.0121374.s006.pdf]

# Standardized Residuals Plot

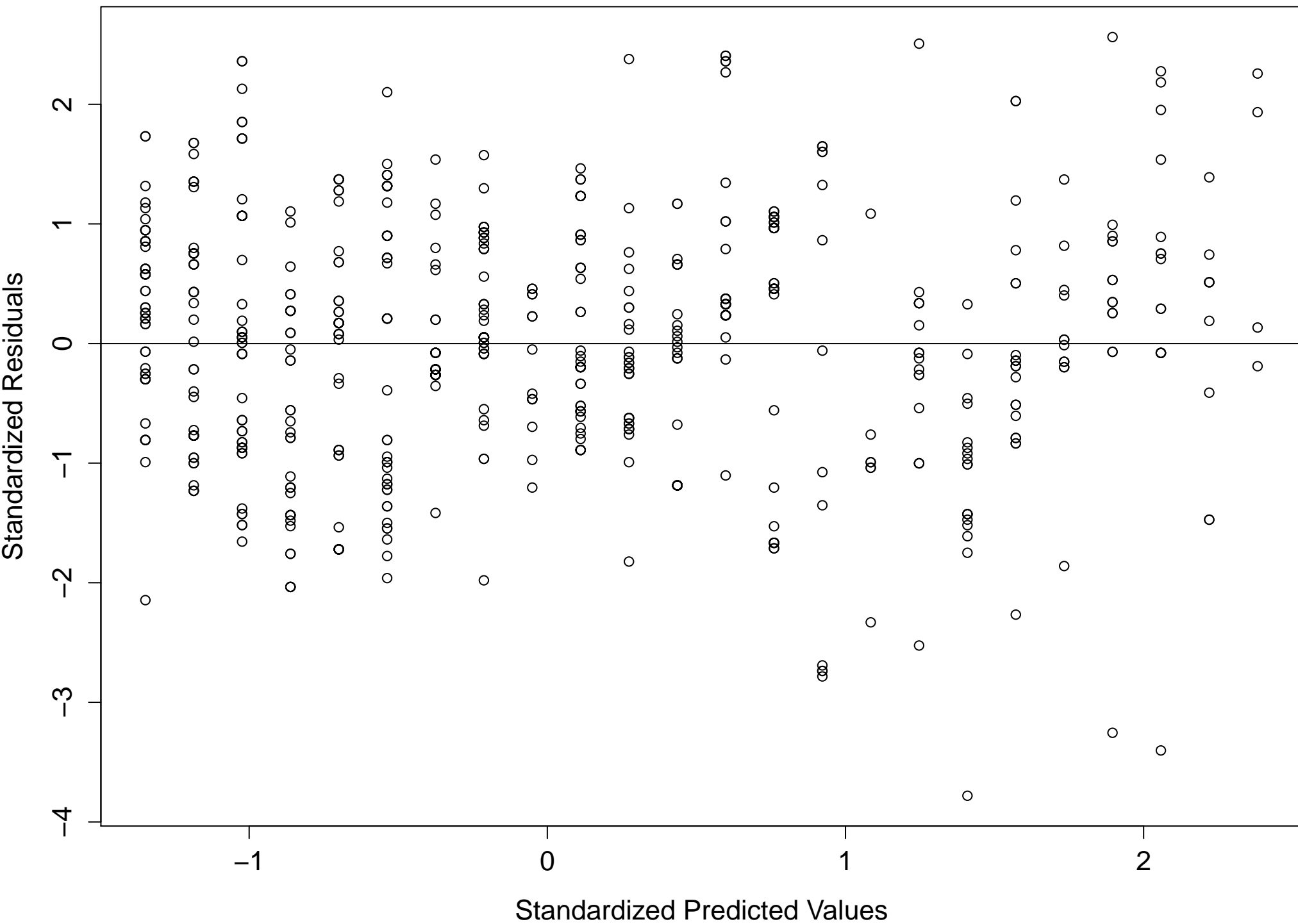

Supplement: S7 Fig — (PDF) [file pone.0121374.s007.pdf]

# Histogram of Standardized Residuals

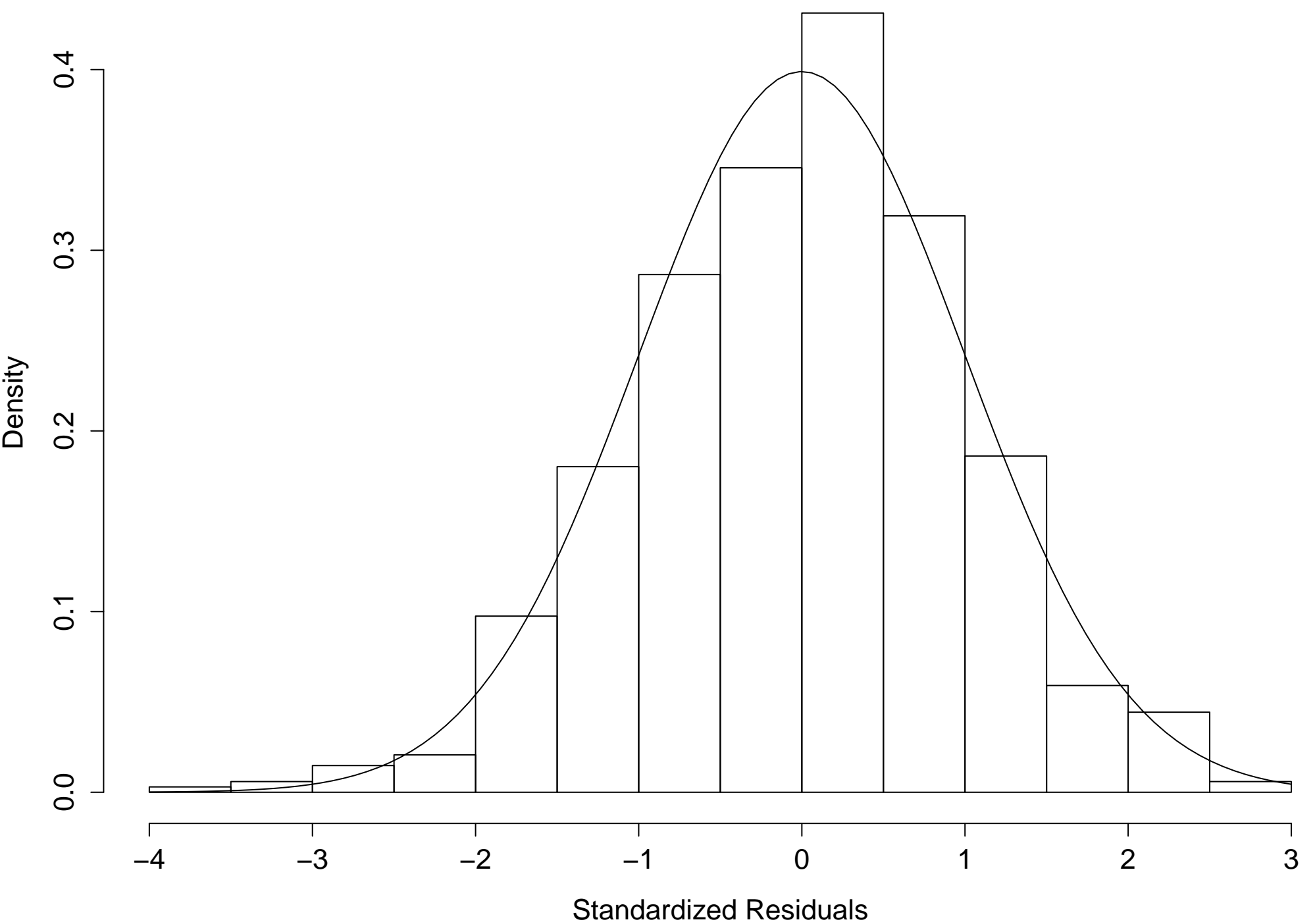

Supplement: S8 Fig — (PDF) [file pone.0121374.s008.pdf]
