# Supplementary material for: Adapting to a Warmer Ocean—Seasonal Shift of Baleen Whale Movements over Three Decades
Source: PLoS One. 2015 Mar 18;10(3):e0121374. doi: 10.1371/journal.pone.0121374 (PMC4364899; doi:10.1371/journal.pone.0121374)
Supplement: S1 File — Arrival and Departure Dates for all Individuals. (PDF) [file pone.0121374.s009.pdf]

Fin Whale Raw Data

Year	Individual	Arrival	Departure
1984	F002	225	225
1984	F012	219	219
1984	F018	231	231
1984	F025	230	232
1984	F032	236	236
1984	F042	219	219
1984	F046	221	230
1984	F047	274	274
1984	F111	238	238
1984	F183	238	283
1984	F389	283	283
1985	F002	200	200
1985	F005	243	243
1985	F012	286	286
1985	F022	253	253
1985	F028	253	253
1985	F031	253	253
1985	F043	249	249
1985	F045	200	200
1985	F046	249	249
1985	F047	198	198
1985	F051	248	249
1985	F079	286	286
1985	F099	200	200
1985	F108	286	286
1986	F002	246	263
1986	F003	247	247
1986	F005	193	194
1986	F012	219	219
1986	F016	276	276
1986	F018	193	211
1986	F019	227	227
1986	F028	263	263
1986	F041	231	260
1986	F043	221	221
1986	F045	231	231
1986	F046	205	222
1986	F051	231	231
1986	F064	247	247
1986	F068	266	266
1986	F070	211	246
1986	F086	246	263
1986	F103	246	246
1986	F118	194	194
1986	F142	266	266
1986	F214	266	266
1987	F001	169	198

Humpback Whale Raw Data

Year	Individual	Arrival	Departure
1987	H002	241	247
1987	H007	248	248
1987	H044	287	301
1987	H129	294	301
1988	H001	260	290
1988	H002	212	214
1988	H006	255	290
1988	H008	255	290
1988	H009	255	290
1988	H022	248	290
1988	H042	235	289
1988	H044	274	290
1988	H129	212	290
1989	H001	241	290
1989	H002	241	298
1989	H004	262	298
1989	H007	249	249
1989	H008	285	285
1989	H009	259	298
1989	H012	241	297
1989	H022	258	297
1989	H042	259	293
1989	H044	249	297
1989	H107	290	299
1989	H109	276	299
1989	H128	169	296
1989	H129	292	292
1989	H137	241	241
1990	H001	263	295
1990	H004	253	263
1990	H006	240	249
1990	H007	260	296
1990	H008	260	296
1990	H009	249	273
1990	H012	297	297
1990	H042	240	295
1990	H044	247	273
1990	H107	249	263
1990	H109	261	261
1990	H128	171	296
1990	H129	253	263
1990	H140	247	261
1991	H001	240	274
1991	H002	241	241
1991	H004	241	241
1991	H007	250	250
1991	H009	237	241

1987	F002	172	187
1987	F005	183	183
1987	F006	164	211
1987	F015	169	198
1987	F017	169	169
1987	F018	184	184
1987	F020	242	242
1987	F022	191	191
1987	F025	198	215
1987	F028	185	191
1987	F032	198	206
1987	F036	184	286
1987	F037	187	192
1987	F042	206	206
1987	F043	206	206
1987	F045	184	185
1987	F046	215	293
1987	F049	286	300
1987	F070	206	206
1987	F078	169	169
1987	F080	285	285
1987	F088	201	203
1987	F099	187	187
1987	F118	198	198
1987	F161	227	227
1987	F163	285	285
1987	F171	242	242
1987	F212	168	168
1987	F213	285	293
1987	F299	201	201
1988	F001	176	176
1988	F002	180	229
1988	F015	216	216
1988	F016	246	246
1988	F018	180	180
1988	F020	208	244
1988	F021	169	180
1988	F022	176	176
1988	F025	186	229
1988	F026	236	236
1988	F032	186	186
1988	F045	180	180
1988	F048	246	246
1988	F052	235	235
1988	F053	229	229
1988	F055	176	176
1988	F056	255	255
1988	F068	229	229
1988	F069	236	236
1991	H012	258	258
1991	H042	270	270
1991	H044	200	232
1991	H103	249	250
1991	H107	237	237
1991	H109	236	250
1991	H140	236	250
1992	H001	235	286
1992	H002	221	287
1992	H004	226	287
1992	H006	233	288
1992	H007	237	277
1992	H008	222	288
1992	H009	283	283
1992	H012	256	295
1992	H042	283	296
1992	H044	250	288
1992	H067	222	272
1992	H069	250	282
1992	H107	235	271
1992	H109	221	248
1992	H128	265	295
1992	H129	228	287
1992	H140	228	270
1992	H141	236	288
1992	H142	236	245
1992	H145	190	288
1992	H146	236	282
1993	H001	219	230
1993	H002	227	236
1993	H004	218	248
1993	H006	217	231
1993	H008	216	236
1993	H009	207	244
1993	H042	205	230
1993	H044	216	244
1993	H067	207	244
1993	H069	245	245
1993	H109	228	248
1993	H128	220	233
1993	H129	207	235
1993	H141	200	245
1993	H144	206	245
1993	H145	156	245
1993	H146	203	234
1993	H150	236	247
1994	H001	235	235
1994	H002	235	246
1994	H006	232	232

1988	F080	255	255
1988	F084	176	224
1988	F088	226	227
1988	F089	180	180
1988	F101	236	245
1988	F118	176	176
1988	F150	216	216
1988	F153	224	224
1988	F154	188	188
1988	F179	246	246
1988	F252	180	180
1989	F002	268	268
1989	F010	229	229
1989	F016	229	258
1989	F025	183	183
1989	F027	182	279
1989	F030	256	258
1989	F033	182	185
1989	F035	241	241
1989	F040	301	301
1989	F042	182	182
1989	F058	182	182
1989	F059	213	213
1989	F060	268	279
1989	F065	213	213
1989	F066	258	258
1989	F071	182	182
1989	F080	182	182
1989	F087	255	255
1989	F093	183	185
1989	F095	258	258
1989	F097	213	213
1989	F101	254	254
1989	F104	183	183
1989	F109	254	254
1989	F110	241	241
1989	F111	279	279
1989	F130	258	279
1989	F137	241	241
1989	F155	241	241
1989	F167	297	297
1989	F179	248	248
1989	F299	254	254
1989	F333	241	241
1989	F351	258	258
1989	F369	182	183
1990	F002	240	240
1990	F025	237	240
1990	F027	207	207
1994	H007	240	240
1994	H008	231	232
1994	H009	215	215
1994	H012	235	235
1994	H033	291	291
1994	H042	244	244
1994	H044	234	246
1994	H067	246	246
1994	H107	231	245
1994	H109	215	235
1994	H129	215	215
1994	H142	182	182
1994	H164	225	225
1994	H166	244	248
1994	H168	244	244
1995	H009	213	215
1995	H042	213	215
1995	H107	219	219
1995	H135	259	259
1995	H141	214	214
1995	H149	214	214
1995	H165	185	185
1996	H002	270	270
1996	H004	233	269
1996	H009	269	269
1996	H022	253	253
1996	H042	205	205
1996	H054	175	175
1996	H056	176	176
1996	H067	175	181
1996	H109	211	211
1996	H141	270	270
1996	H163	269	269
1996	H165	174	174
1996	H277	263	263
1997	H002	255	256
1997	H004	243	243
1997	H006	242	254
1997	H007	255	255
1997	H008	256	256
1997	H009	196	280
1997	H012	257	257
1997	H022	244	256
1997	H042	254	260
1997	H044	197	286
1997	H067	254	282
1997	H071	254	256
1997	H109	256	257
1997	H128	207	255

1990	F029	237	237
1990	F033	238	240
1990	F036	216	216
1990	F043	166	166
1990	F060	263	263
1990	F080	282	282
1990	F084	249	249
1990	F087	236	249
1990	F101	249	249
1990	F113	217	281
1990	F114	240	240
1990	F116	249	249
1990	F118	247	247
1990	F119	247	247
1990	F120	249	249
1990	F121	246	246
1990	F123	249	249
1990	F125	217	217
1990	F140	218	218
1990	F141	236	236
1990	F147	188	188
1990	F150	281	281
1990	F157	249	249
1990	F180	281	281
1990	F320	277	277
1991	F001	194	229
1991	F018	199	245
1991	F027	254	254
1991	F030	212	212
1991	F036	186	250
1991	F041	211	211
1991	F042	241	245
1991	F069	258	258
1991	F087	249	249
1991	F093	199	200
1991	F099	237	237
1991	F109	185	185
1991	F150	229	231
1991	F157	200	231
1991	F165	246	246
1991	F166	242	242
1991	F167	229	242
1991	F173	185	185
1991	F174	250	250
1991	F176	237	237
1991	F180	237	237
1991	F183	199	227
1991	F185	236	236
1991	F187	200	200
1997	H141	243	243
1997	H144	197	286
1997	H146	256	256
1997	H147	243	243
1997	H149	196	254
1997	H150	254	254
1997	H151	197	257
1997	H152	221	221
1997	H163	257	257
1997	H164	244	286
1997	H165	254	260
1997	H166	243	256
1997	H168	243	254
1997	H228	200	257
1997	H263	197	197
1997	H275	256	256
1997	H277	197	197
1997	H286	254	260
1997	H288	254	256
1997	H329	256	256
1997	H379	243	254
1998	H001	208	208
1998	H002	239	240
1998	H004	240	286
1998	H006	237	282
1998	H007	237	240
1998	H009	237	282
1998	H012	237	239
1998	H044	237	281
1998	H056	249	249
1998	H067	284	284
1998	H069	239	239
1998	H071	254	254
1998	H107	237	281
1998	H109	239	289
1998	H118	240	290
1998	H128	283	283
1998	H129	281	281
1998	H135	239	280
1998	H140	240	282
1998	H141	239	283
1998	H144	239	290
1998	H145	233	240
1998	H147	239	240
1998	H150	240	240
1998	H151	240	254
1998	H152	283	283
1998	H164	261	261
1998	H165	254	254

1991	F188	199	199	1998	H228	239	240
1991	F190	237	237	1998	H263	284	291
1991	F192	258	258	1998	H275	237	240
1991	F196	258	258	1998	H277	237	291
1991	F197	212	212	1998	H288	239	283
1991	F198	240	250	1998	H329	237	237
1991	F200	229	229	1998	H379	239	253
1991	F201	236	236	1998	H405	239	283
1991	F204	199	212	1998	H406	229	239
1991	F207	184	184	1998	H457	239	240
1991	F208	199	199	1998	H458	237	282
1991	F212	242	242	1999	H002	205	270
1992	F001	227	227	1999	H004	233	256
1992	F012	239	239	1999	H007	205	269
1992	F014	217	217	1999	H008	245	270
1992	F019	196	196	1999	H009	245	270
1992	F021	208	208	1999	H012	245	268
1992	F027	235	235	1999	H042	205	261
1992	F028	192	192	1999	H044	234	255
1992	F029	208	208	1999	H067	205	267
1992	F033	179	235	1999	H069	228	255
1992	F036	214	228	1999	H071	205	205
1992	F039	273	273	1999	H100	246	251
1992	F044	181	181	1999	H109	205	269
1992	F045	190	190	1999	H128	236	256
1992	F046	226	227	1999	H129	232	256
1992	F049	204	204	1999	H140	205	268
1992	F053	227	227	1999	H141	229	270
1992	F056	288	288	1999	H144	256	256
1992	F066	273	273	1999	H145	205	262
1992	F084	202	208	1999	H146	228	268
1992	F086	227	227	1999	H147	205	268
1992	F087	282	282	1999	H150	256	268
1992	F093	204	235	1999	H151	205	268
1992	F099	217	217	1999	H165	216	216
1992	F109	179	280	1999	H166	245	270
1992	F116	204	235	1999	H168	228	268
1992	F118	271	271	1999	H275	230	267
1992	F147	222	227	1999	H277	205	270
1992	F150	271	271	1999	H329	205	205
1992	F166	169	169	1999	H406	231	270
1992	F167	204	204	2000	H002	239	241
1992	F177	208	208	2000	H004	216	258
1992	F179	226	239	2000	H006	254	254
1992	F180	269	269	2000	H007	214	283
1992	F185	194	194	2000	H042	216	271
1992	F187	204	204	2000	H044	190	271
1992	F193	181	181	2000	H067	214	243
1992	F204	202	202	2000	H069	215	236

1992	F210	181	181	2000	H109	215	241
1992	F212	227	306	2000	H128	281	281
1992	F216	204	204	2000	H129	216	241
1992	F222	195	195	2000	H135	224	228
1992	F223	202	204	2000	H140	216	225
1992	F224	181	195	2000	H141	225	300
1992	F225	226	227	2000	H144	213	300
1992	F226	181	181	2000	H146	225	254
1992	F227	181	181	2000	H147	224	273
1992	F230	269	282	2000	H150	246	246
1992	F232	227	227	2000	H151	232	239
1992	F234	271	271	2000	H163	243	273
1992	F235	226	282	2000	H164	236	273
1992	F236	273	273	2000	H166	224	283
1992	F243	269	269	2000	H168	236	254
1992	F245	195	227	2000	H212	226	226
1992	F248	180	217	2000	H228	233	246
1992	F252	179	282	2000	H263	225	239
1992	F253	235	235	2000	H277	208	258
1992	F254	227	227	2000	H286	228	228
1992	F255	217	282	2000	H329	214	283
1992	F259	235	235	2000	H379	227	235
1992	F261	227	227	2000	H405	227	282
1992	F263	179	179	2000	H406	214	243
1992	F264	238	238	2000	H459	226	228
1992	F266	271	271	2001	H002	255	294
1992	F267	269	269	2001	H004	209	263
1992	F271	271	271	2001	H006	215	266
1992	F273	271	271	2001	H007	211	211
1992	F274	269	269	2001	H008	224	271
1992	F276	271	271	2001	H009	255	263
1992	F277	227	227	2001	H012	260	262
1992	F280	202	202	2001	H044	213	262
1992	F281	202	202	2001	H067	240	267
1992	F282	217	217	2001	H069	216	259
1992	F283	275	277	2001	H107	216	259
1992	F285	235	235	2001	H109	248	294
1992	F286	227	271	2001	H118	242	284
1992	F291	192	192	2001	H128	224	293
1992	F292	202	202	2001	H129	248	262
1992	F298	269	269	2001	H140	216	296
1992	F299	217	288	2001	H141	209	294
1992	F300	269	269	2001	H144	216	294
1992	F310	235	235	2001	H145	212	262
1992	F311	273	273	2001	H146	234	263
1992	F315	235	235	2001	H147	224	268
1992	F316	235	235	2001	H150	234	262
1992	F318	202	204	2001	H151	224	262
1992	F320	271	282	2001	H152	248	248

1992	F386	204	204	2001	H156	255	255
1993	F001	200	220	2001	H164	242	262
1993	F002	170	187	2001	H165	209	272
1993	F003	205	205	2001	H166	247	262
1993	F004	245	245	2001	H168	227	227
1993	F012	170	231	2001	H228	217	268
1993	F016	228	228	2001	H263	224	224
1993	F018	187	187	2001	H275	209	296
1993	F025	187	187	2001	H277	226	227
1993	F027	200	216	2001	H286	221	221
1993	F028	186	186	2001	H379	242	262
1993	F029	187	187	2001	H405	258	258
1993	F032	219	231	2001	H406	224	224
1993	F033	247	247	2001	H456	247	296
1993	F035	245	245	2001	H458	255	255
1993	F036	208	236	2001	H472	225	295
1993	F039	230	230	2001	H485	217	272
1993	F042	162	162	2001	H491	224	224
1993	F045	230	230	2001	H493	221	260
1993	F053	207	207	2001	H494	258	262
1993	F059	207	207	2002	H009	217	240
1993	F060	227	228	2002	H129	236	237
1993	F068	200	200	2002	H141	206	207
1993	F080	228	230	2002	H146	226	237
1993	F084	231	236	2002	H150	236	236
1993	F086	156	156	2002	H164	237	257
1993	F093	200	228	2002	H166	237	237
1993	F101	219	230	2002	H228	217	240
1993	F110	227	230	2002	H277	201	260
1993	F119	207	207	2002	H329	218	218
1993	F147	228	231	2002	H461	217	238
1993	F155	228	228	2002	H472	232	240
1993	F176	156	156	2002	H485	232	240
1993	F183	172	217	2002	H531	212	213
1993	F188	156	162	2003	H002	230	245
1993	F197	230	230	2003	H004	231	256
1993	F198	206	247	2003	H006	243	260
1993	F201	207	207	2003	H007	227	249
1993	F204	186	218	2003	H008	233	233
1993	F207	200	228	2003	H009	227	267
1993	F212	200	230	2003	H012	246	261
1993	F224	206	247	2003	H035	260	260
1993	F227	216	216	2003	H042	244	244
1993	F230	247	247	2003	H044	211	256
1993	F243	228	236	2003	H067	226	255
1993	F252	170	172	2003	H069	246	256
1993	F263	220	231	2003	H071	246	256
1993	F280	200	231	2003	H107	247	260
1993	F297	200	200	2003	H109	205	267

1993	F298	207	207	2003	H128	225	246
1993	F304	200	200	2003	H129	205	259
1993	F316	219	230	2003	H133	246	256
1993	F322	200	200	2003	H140	227	260
1993	F324	228	247	2003	H141	224	255
1993	F333	200	219	2003	H144	246	256
1993	F334	200	236	2003	H146	238	255
1993	F335	228	230	2003	H147	224	255
1993	F338	200	200	2003	H150	183	229
1993	F339	206	206	2003	H151	214	253
1993	F341	269	269	2003	H163	247	268
1993	F343	172	172	2003	H164	243	268
1993	F344	156	231	2003	H165	232	256
1993	F345	230	231	2003	H166	227	255
1993	F346	206	228	2003	H168	247	260
1993	F348	156	156	2003	H228	227	257
1993	F349	228	230	2003	H234	254	256
1993	F350	206	207	2003	H275	233	262
1993	F351	217	220	2003	H277	212	258
1993	F355	230	230	2003	H288	243	257
1993	F358	247	247	2003	H329	245	256
1993	F359	205	234	2003	H379	224	262
1993	F369	217	217	2003	H406	227	255
1993	F375	247	269	2003	H459	233	249
1993	F376	206	206	2003	H460	245	260
1994	F003	225	225	2003	H461	227	267
1994	F036	182	221	2003	H472	227	257
1994	F039	246	246	2003	H485	245	256
1994	F097	225	244	2003	H493	226	255
1994	F099	186	186	2003	H509	224	255
1994	F163	218	219	2003	H531	225	255
1994	F198	225	244	2004	H002	219	261
1994	F207	246	246	2004	H006	229	254
1994	F232	231	231	2004	H007	220	254
1994	F263	244	246	2004	H008	223	242
1994	F267	225	225	2004	H009	217	261
1994	F286	244	248	2004	H044	219	230
1994	F299	225	285	2004	H067	250	255
1994	F316	244	246	2004	H069	238	266
1994	F382	244	244	2004	H109	220	223
1994	F383	234	234	2004	H118	239	266
1994	F384	225	225	2004	H128	248	266
1994	F385	248	248	2004	H129	219	248
1994	F388	216	225	2004	H140	219	267
1995	F002	208	208	2004	H141	220	266
1995	F036	208	208	2004	H146	220	266
1995	F095	197	197	2004	H147	223	223
1995	F101	208	208	2004	H150	258	267
1995	F150	216	252	2004	H164	242	262

1995	F159	208	208	2004	H228	219	267
1995	F183	191	198	2004	H277	220	266
1995	F276	252	252	2004	H329	219	229
1995	F299	197	208	2004	H379	219	264
1995	F316	185	185	2004	H405	219	262
1995	F412	215	215	2004	H406	229	265
1995	F414	259	259	2004	H461	220	234
1995	F416	197	218	2004	H472	223	242
1996	F018	205	248	2004	H492	223	263
1996	F021	210	210	2004	H493	194	263
1996	F028	268	268	2004	H502	220	266
1996	F087	172	172	2004	H509	223	264
1996	F198	205	205	2004	H531	219	239
1996	F217	205	205	2005	H002	206	252
1996	F218	172	172	2005	H004	254	256
1996	F244	211	211	2005	H006	252	305
1996	F245	180	180	2005	H007	206	207
1996	F275	214	214	2005	H008	228	255
1996	F299	193	193	2005	H009	202	252
1996	F314	205	205	2005	H012	239	239
1997	F002	214	214	2005	H042	238	258
1997	F036	260	260	2005	H044	198	232
1997	F116	225	225	2005	H067	256	257
1997	F148	174	174	2005	H069	252	254
1997	F163	218	220	2005	H107	188	229
1997	F175	254	254	2005	H109	190	269
1997	F243	221	221	2005	H118	191	239
1997	F268	176	176	2005	H128	228	256
1997	F285	169	254	2005	H129	202	242
1997	F291	174	174	2005	H140	254	256
1997	F294	169	169	2005	H141	203	256
1997	F299	221	221	2005	H144	198	256
1997	F305	221	221	2005	H146	197	256
1997	F310	254	254	2005	H150	249	249
1997	F335	272	272	2005	H151	202	236
1997	F376	272	272	2005	H163	269	291
1997	F418	224	224	2005	H166	252	256
1997	F429	174	174	2005	H228	228	271
1997	F435	244	244	2005	H263	228	263
1998	F025	212	212	2005	H275	201	271
1998	F028	216	216	2005	H277	181	206
1998	F037	225	225	2005	H286	185	253
1998	F041	219	219	2005	H288	243	243
1998	F045	217	217	2005	H329	194	242
1998	F099	218	218	2005	H379	243	271
1998	F224	216	216	2005	H405	252	254
1998	F299	225	225	2005	H406	215	216
1998	F301	239	239	2005	H456	198	207
1998	F388	282	282	2005	H459	239	239

1998	F399	219	219	2005	H460	254	256
1998	F412	218	218	2005	H461	190	252
1998	F422	216	216	2005	H472	239	256
1998	F426	212	225	2005	H485	243	258
1998	F429	179	179	2005	H487	252	254
1998	F441	218	218	2005	H492	239	256
1998	F450	282	282	2005	H493	228	254
1998	F451	282	282	2005	H494	243	256
1998	F455	218	218	2005	H502	194	255
1998	F457	239	239	2005	H509	243	263
1999	F016	231	231	2005	H531	197	258
1999	F025	228	228	2005	H555	199	256
1999	F039	178	178	2005	H648	252	256
1999	F049	232	232	2006	H006	203	239
1999	F054	178	178	2006	H008	226	229
1999	F060	231	232	2006	H009	202	238
1999	F072	176	205	2006	H012	252	260
1999	F098	244	244	2006	H042	226	239
1999	F129	173	173	2006	H044	203	213
1999	F139	251	251	2006	H107	228	230
1999	F143	216	216	2006	H109	185	203
1999	F175	232	232	2006	H129	185	185
1999	F176	232	232	2006	H140	185	185
1999	F207	216	216	2006	H141	185	206
1999	F245	178	178	2006	H144	250	250
1999	F299	232	232	2006	H146	185	216
1999	F358	215	216	2006	H150	226	253
1999	F416	232	232	2006	H163	230	250
1999	F423	178	178	2006	H166	223	237
1999	F449	178	178	2006	H168	224	224
1999	F457	228	232	2006	H228	203	270
2000	F002	194	212	2006	H274	228	237
2000	F009	248	252	2006	H275	224	239
2000	F018	188	242	2006	H277	202	223
2000	F025	190	248	2006	H286	185	266
2000	F028	210	256	2006	H288	237	239
2000	F038	227	227	2006	H406	226	248
2000	F039	176	203	2006	H420	237	239
2000	F045	213	241	2006	H437	252	269
2000	F049	204	204	2006	H456	189	203
2000	F060	181	181	2006	H458	252	270
2000	F067	214	214	2006	H460	239	239
2000	F072	213	215	2006	H461	250	250
2000	F087	190	247	2006	H485	226	248
2000	F097	242	248	2006	H491	226	226
2000	F099	192	225	2006	H492	216	216
2000	F100	258	258	2006	H493	215	218
2000	F102	242	247	2006	H502	248	248
2000	F115	188	188	2006	H529	230	231

2000	F119	242	242	2006	H531	185	218
2000	F134	213	213	2006	H584	224	239
2000	F143	181	181	2007	H002	190	220
2000	F170	181	212	2007	H006	190	227
2000	F175	241	255	2007	H008	207	213
2000	F176	181	181	2007	H042	209	227
2000	F183	195	195	2007	H044	197	227
2000	F187	190	192	2007	H107	220	227
2000	F198	204	204	2007	H109	177	218
2000	F217	188	188	2007	H118	204	224
2000	F237	214	214	2007	H140	188	197
2000	F238	186	186	2007	H141	183	245
2000	F247	210	213	2007	H144	195	218
2000	F274	228	228	2007	H146	223	223
2000	F284	252	252	2007	H151	218	227
2000	F285	176	213	2007	H163	243	243
2000	F291	176	247	2007	H168	195	223
2000	F299	189	196	2007	H212	230	230
2000	F304	181	181	2007	H228	190	243
2000	F323	241	241	2007	H263	227	230
2000	F412	300	300	2007	H275	209	227
2001	F002	246	246	2007	H277	190	224
2001	F018	163	251	2007	H379	190	234
2001	F028	166	284	2007	H405	227	227
2001	F029	308	308	2007	H406	189	234
2001	F038	217	217	2007	H456	227	227
2001	F039	189	225	2007	H458	235	235
2001	F045	163	163	2007	H459	227	227
2001	F067	224	228	2007	H460	227	227
2001	F087	296	296	2007	H461	205	235
2001	F098	211	217	2007	H472	218	243
2001	F099	242	242	2007	H485	194	195
2001	F100	217	217	2007	H492	195	243
2001	F101	199	199	2007	H493	183	210
2001	F102	189	228	2007	H502	204	243
2001	F115	199	201	2007	H509	177	230
2001	F116	216	224	2007	H531	177	218
2001	F119	211	211	2007	H546	177	227
2001	F125	240	240	2007	H573	223	230
2001	F146	217	248	2007	H584	245	245
2001	F170	217	218	2007	H591	230	230
2001	F172	216	217	2008	H002	266	271
2001	F175	218	218	2008	H007	222	271
2001	F183	200	294	2008	H008	260	271
2001	F185	197	197	2008	H009	227	271
2001	F200	228	228	2008	H012	243	271
2001	F205	217	217	2008	H042	201	271
2001	F207	216	217	2008	H044	187	271
2001	F226	199	212	2008	H069	220	228

2001	F238	224	224	2008	H102	204	271
2001	F251	212	240	2008	H107	202	246
2001	F252	163	163	2008	H109	201	271
2001	F258	224	224	2008	H128	206	266
2001	F276	248	248	2008	H129	221	271
2001	F277	255	255	2008	H140	187	271
2001	F280	216	217	2008	H141	190	261
2001	F286	199	260	2008	H144	200	246
2001	F288	218	218	2008	H146	201	271
2001	F291	166	212	2008	H150	221	236
2001	F299	161	308	2008	H163	257	271
2001	F304	242	255	2008	H164	222	243
2001	F307	201	202	2008	H166	222	236
2001	F312	294	294	2008	H228	189	271
2001	F318	284	284	2008	H263	220	271
2001	F344	189	189	2008	H275	218	218
2001	F351	216	217	2008	H277	201	271
2001	F383	212	212	2008	H286	257	257
2001	F410	212	212	2008	H288	210	210
2002	F002	182	236	2008	H379	243	243
2002	F018	237	274	2008	H405	271	271
2002	F028	201	201	2008	H406	260	260
2002	F034	189	201	2008	H420	204	210
2002	F039	222	222	2008	H437	243	271
2002	F045	191	216	2008	H456	271	271
2002	F054	222	222	2008	H458	246	261
2002	F067	201	202	2008	H459	243	271
2002	F087	217	217	2008	H460	243	243
2002	F092	201	201	2008	H461	257	261
2002	F099	237	237	2008	H485	200	266
2002	F100	222	222	2008	H493	184	246
2002	F115	201	224	2008	H502	218	228
2002	F124	232	232	2008	H529	221	236
2002	F130	207	208	2008	H531	218	257
2002	F131	201	222	2008	H546	243	271
2002	F137	232	232	2008	H573	221	221
2002	F139	218	221	2008	H584	206	261
2002	F143	202	202	2008	H586	202	227
2002	F144	207	207	2008	H591	235	271
2002	F150	207	207	2008	H672	189	266
2002	F152	206	218	2008	H738	224	240
2002	F165	202	202	2009	H002	219	241
2002	F175	232	232	2009	H004	228	260
2002	F183	182	201	2009	H006	202	248
2002	F199	202	202	2009	H008	235	253
2002	F207	201	201	2009	H009	202	255
2002	F225	250	250	2009	H012	248	248
2002	F226	206	206	2009	H042	209	260
2002	F235	232	232	2009	H044	235	235

2002	F280	207	250	2009	H069	202	204
2002	F299	182	237	2009	H107	248	248
2002	F303	201	201	2009	H109	248	260
2002	F304	201	232	2009	H118	210	210
2002	F320	201	240	2009	H128	253	255
2002	F337	230	250	2009	H129	198	260
2002	F345	206	207	2009	H140	228	229
2002	F402	246	246	2009	H141	255	255
2003	F002	173	274	2009	H144	233	255
2003	F016	217	217	2009	H146	214	253
2003	F018	214	232	2009	H150	235	255
2003	F025	274	274	2009	H151	247	247
2003	F028	274	274	2009	H163	255	255
2003	F034	173	173	2009	H164	214	237
2003	F039	204	246	2009	H168	233	248
2003	F040	244	246	2009	H228	192	241
2003	F099	205	205	2009	H263	202	203
2003	F100	214	215	2009	H274	228	260
2003	F102	214	226	2009	H277	198	227
2003	F115	210	221	2009	H288	193	205
2003	F131	212	212	2009	H379	202	204
2003	F135	221	221	2009	H405	236	263
2003	F139	221	244	2009	H406	197	248
2003	F143	221	221	2009	H437	235	264
2003	F146	214	214	2009	H456	202	260
2003	F166	221	221	2009	H458	197	253
2003	F181	226	226	2009	H460	226	226
2003	F183	250	250	2009	H472	233	260
2003	F207	204	211	2009	H485	235	248
2003	F228	218	221	2009	H491	223	249
2003	F241	246	246	2009	H492	202	264
2003	F242	244	244	2009	H493	203	263
2003	F252	214	226	2009	H509	192	263
2003	F280	246	246	2009	H531	192	202
2003	F282	227	250	2009	H586	202	203
2003	F291	205	274	2009	H591	235	241
2003	F293	246	246	2010	H002	238	243
2003	F299	214	217	2010	H004	200	238
2003	F303	246	246	2010	H006	238	246
2003	F304	245	246	2010	H007	224	246
2003	F318	215	226	2010	H008	211	227
2003	F348	195	205	2010	H009	238	243
2003	F389	214	214	2010	H012	242	242
2003	F411	256	256	2010	H042	211	231
2004	F002	230	258	2010	H044	200	226
2004	F004	218	238	2010	H069	255	261
2004	F008	229	251	2010	H107	213	261
2004	F024	219	250	2010	H109	255	255
2004	F028	194	243	2010	H128	236	246

2004	F029	207	207
2004	F034	211	211
2004	F035	238	248
2004	F039	200	200
2004	F045	211	247
2004	F049	194	209
2004	F054	213	250
2004	F087	194	250
2004	F092	219	253
2004	F096	253	253
2004	F098	267	267
2004	F099	219	238
2004	F100	194	265
2004	F101	196	219
2004	F102	230	258
2004	F109	196	196
2004	F115	194	250
2004	F119	208	230
2004	F121	248	265
2004	F122	194	256
2004	F124	219	247
2004	F132	219	260
2004	F135	219	230
2004	F137	215	265
2004	F138	209	231
2004	F139	247	248
2004	F143	194	229
2004	F144	209	250
2004	F146	229	250
2004	F148	218	250
2004	F149	223	251
2004	F150	194	248
2004	F151	219	267
2004	F159	207	207
2004	F162	230	238
2004	F165	196	210
2004	F170	266	266
2004	F172	194	196
2004	F173	219	249
2004	F181	223	223
2004	F183	194	231
2004	F187	200	219
2004	F189	194	196
2004	F191	207	223
2004	F192	229	230
2004	F193	230	265
2004	F194	215	248
2004	F199	230	230
2004	F203	219	248

2010	H129	211	213
2010	H140	212	226
2010	H141	200	231
2010	H144	227	231
2010	H146	227	246
2010	H150	231	231
2010	H151	230	246
2010	H163	216	236
2010	H166	231	238
2010	H168	221	261
2010	H228	200	255
2010	H263	211	236
2010	H277	196	227
2010	H286	236	236
2010	H379	238	261
2010	H405	230	261
2010	H406	240	243
2010	H437	230	231
2010	H456	221	261
2010	H458	222	238
2010	H459	236	238
2010	H461	212	255
2010	H472	222	255
2010	H485	231	244
2010	H491	235	238
2010	H492	224	255
2010	H493	203	255
2010	H502	223	242
2010	H509	236	238
2010	H531	230	244
2010	H546	230	255
2010	H573	243	246
2010	H586	216	242
2010	H591	211	213
2010	H607	230	236
2010	H624	211	255
2010	H640	230	246
2010	H670	246	246
2010	H671	223	261
2010	H676	223	236
2010	H678	222	255
2010	H683	171	261

2004	F205	209	253
2004	F212	196	260
2004	F217	230	230
2004	F219	219	219
2004	F221	219	230
2004	F222	219	238
2004	F225	239	253
2004	F227	230	230
2004	F228	196	196
2004	F229	189	194
2004	F231	230	230
2004	F236	224	224
2004	F237	208	229
2004	F238	194	194
2004	F242	219	220
2004	F245	194	208
2004	F247	266	266
2004	F252	210	260
2004	F258	230	230
2004	F268	194	194
2004	F282	213	213
2004	F284	251	251
2004	F292	194	219
2004	F297	210	223
2004	F299	193	266
2004	F301	238	250
2004	F304	219	267
2004	F305	215	265
2004	F318	215	250
2004	F320	211	248
2004	F329	229	229
2004	F349	219	238
2004	F351	218	251
2004	F352	220	253
2004	F370	219	219
2004	F382	194	251
2004	F384	247	249
2004	F391	219	219
2004	F400	210	210
2004	F410	194	194
2004	F412	209	209
2004	F413	209	215
2004	F423	193	193
2004	F462	210	230
2004	F474	230	240
2005	F002	228	228
2005	F004	187	188
2005	F008	170	228
2005	F016	169	221

2005	F019	190	192
2005	F024	181	228
2005	F028	204	204
2005	F039	212	221
2005	F045	174	221
2005	F049	207	236
2005	F054	188	201
2005	F059	204	204
2005	F071	185	185
2005	F084	177	185
2005	F085	170	181
2005	F087	174	174
2005	F092	207	207
2005	F098	181	216
2005	F099	174	181
2005	F100	174	221
2005	F101	187	192
2005	F102	194	221
2005	F113	181	216
2005	F115	192	221
2005	F119	174	228
2005	F121	178	193
2005	F125	215	216
2005	F130	215	215
2005	F146	197	214
2005	F149	201	202
2005	F150	177	190
2005	F151	170	236
2005	F159	201	202
2005	F165	181	181
2005	F167	190	190
2005	F170	178	190
2005	F172	174	181
2005	F173	187	228
2005	F175	192	202
2005	F181	181	228
2005	F182	177	187
2005	F183	181	207
2005	F186	214	214
2005	F187	181	181
2005	F189	177	188
2005	F192	170	188
2005	F193	206	258
2005	F195	177	193
2005	F199	188	189
2005	F200	187	263
2005	F203	192	192
2005	F207	221	221
2005	F212	202	202

2005	F222	194	194
2005	F225	239	239
2005	F227	197	202
2005	F228	181	193
2005	F229	177	181
2005	F231	201	202
2005	F233	185	187
2005	F235	214	216
2005	F236	194	194
2005	F237	206	206
2005	F238	170	216
2005	F239	194	216
2005	F242	204	228
2005	F245	175	197
2005	F247	197	221
2005	F248	214	221
2005	F249	220	221
2005	F252	185	187
2005	F257	239	239
2005	F265	185	216
2005	F268	187	239
2005	F277	181	181
2005	F280	187	203
2005	F282	187	187
2005	F285	189	190
2005	F291	181	216
2005	F292	174	189
2005	F299	213	221
2005	F304	189	254
2005	F310	236	236
2005	F318	178	193
2005	F320	178	188
2005	F323	215	215
2005	F343	199	199
2005	F345	212	213
2005	F351	177	190
2005	F352	243	243
2005	F358	214	214
2005	F360	204	204
2005	F369	216	216
2005	F386	181	228
2005	F389	181	181
2005	F401	197	199
2005	F402	216	216
2005	F407	185	185
2005	F412	197	215
2005	F413	206	254
2005	F423	177	177
2005	F457	238	238

2005	F461	188	188
2006	F002	250	259
2006	F004	212	216
2006	F024	216	248
2006	F025	185	239
2006	F028	207	231
2006	F029	224	244
2006	F035	212	216
2006	F037	240	248
2006	F039	205	234
2006	F040	215	234
2006	F045	180	229
2006	F049	201	240
2006	F050	175	175
2006	F059	185	217
2006	F066	230	237
2006	F071	175	248
2006	F084	217	232
2006	F085	255	255
2006	F087	207	255
2006	F091	258	260
2006	F098	201	233
2006	F099	189	238
2006	F100	185	217
2006	F101	216	224
2006	F102	213	244
2006	F104	175	194
2006	F109	206	206
2006	F113	216	234
2006	F119	237	239
2006	F121	207	207
2006	F122	184	184
2006	F125	191	191
2006	F126	238	239
2006	F132	228	228
2006	F139	217	217
2006	F146	185	255
2006	F148	233	233
2006	F151	216	255
2006	F155	189	233
2006	F160	235	237
2006	F162	185	196
2006	F166	240	240
2006	F170	212	216
2006	F175	175	238
2006	F176	228	228
2006	F183	213	216
2006	F191	266	266
2006	F195	212	234

2006	F207	191	238
2006	F230	205	213
2006	F237	191	216
2006	F238	221	248
2006	F241	269	269
2006	F244	212	215
2006	F245	176	193
2006	F247	221	270
2006	F248	184	184
2006	F251	201	201
2006	F253	206	217
2006	F265	176	176
2006	F267	206	233
2006	F268	167	167
2006	F269	175	213
2006	F272	206	212
2006	F273	248	248
2006	F274	233	233
2006	F277	217	217
2006	F278	189	202
2006	F279	175	185
2006	F280	193	202
2006	F282	185	191
2006	F284	185	193
2006	F288	175	260
2006	F289	189	189
2006	F290	173	191
2006	F291	177	221
2006	F292	212	238
2006	F297	206	234
2006	F299	205	234
2006	F300	240	260
2006	F301	212	255
2006	F303	196	244
2006	F304	175	196
2006	F306	191	238
2006	F307	185	221
2006	F308	191	266
2006	F309	201	202
2006	F312	201	202
2006	F313	255	270
2006	F317	212	234
2006	F318	216	255
2006	F319	205	234
2006	F320	207	207
2006	F321	175	216
2006	F323	223	240
2006	F325	238	255
2006	F326	207	255

2006	F327	233	233
2006	F328	212	228
2006	F330	233	240
2006	F331	248	248
2006	F332	233	234
2006	F336	228	231
2006	F337	233	240
2006	F341	201	217
2006	F345	196	216
2006	F346	248	248
2006	F348	221	253
2006	F351	175	259
2006	F352	206	218
2006	F354	230	238
2006	F356	238	238
2006	F357	215	215
2006	F358	233	253
2006	F359	240	255
2006	F360	212	239
2006	F361	238	238
2006	F362	228	232
2006	F363	202	213
2006	F364	196	214
2006	F365	185	201
2006	F366	233	233
2006	F368	238	238
2006	F369	194	202
2006	F370	212	216
2006	F371	233	239
2006	F372	191	240
2006	F374	248	248
2006	F377	176	176
2006	F378	176	192
2006	F380	238	238
2006	F381	196	207
2006	F386	185	255
2006	F388	224	266
2006	F389	213	232
2006	F390	229	229
2006	F400	207	233
2006	F401	248	270
2006	F405	234	234
2006	F410	221	255
2006	F411	233	238
2006	F412	175	217
2006	F413	189	189
2006	F421	232	238
2006	F426	238	238
2006	F430	238	238

2006	F432	238	238
2006	F437	234	270
2006	F439	248	270
2006	F440	234	234
2006	F443	173	173
2006	F455	173	270
2006	F456	229	229
2006	F471	213	217
2007	F002	226	270
2007	F008	195	199
2007	F016	182	195
2007	F025	199	199
2007	F028	227	270
2007	F029	183	227
2007	F037	189	227
2007	F039	190	195
2007	F045	164	205
2007	F049	202	202
2007	F059	195	197
2007	F072	234	234
2007	F084	213	220
2007	F091	183	234
2007	F092	189	223
2007	F098	199	199
2007	F099	190	227
2007	F100	183	183
2007	F101	213	234
2007	F102	184	252
2007	F109	186	195
2007	F113	194	194
2007	F119	183	195
2007	F122	218	223
2007	F124	175	214
2007	F146	210	226
2007	F148	190	223
2007	F149	243	243
2007	F150	183	243
2007	F155	213	249
2007	F159	227	227
2007	F165	184	218
2007	F167	185	230
2007	F170	223	234
2007	F172	182	234
2007	F173	185	261
2007	F175	185	227
2007	F176	220	220
2007	F181	176	200
2007	F183	199	252
2007	F186	182	190

2007	F187	220	234
2007	F189	190	227
2007	F191	190	218
2007	F193	175	223
2007	F195	184	230
2007	F200	189	227
2007	F203	182	213
2007	F207	226	270
2007	F221	190	190
2007	F225	185	227
2007	F227	243	243
2007	F231	200	200
2007	F235	226	226
2007	F238	182	184
2007	F241	186	243
2007	F242	190	190
2007	F243	235	243
2007	F245	175	175
2007	F247	185	261
2007	F251	226	226
2007	F252	235	235
2007	F253	200	200
2007	F258	185	186
2007	F265	176	213
2007	F267	211	240
2007	F268	195	243
2007	F269	195	195
2007	F274	190	190
2007	F285	210	243
2007	F291	177	252
2007	F297	190	223
2007	F299	190	190
2007	F300	190	190
2007	F303	186	243
2007	F304	195	213
2007	F305	189	218
2007	F310	226	230
2007	F312	199	243
2007	F316	189	226
2007	F320	185	200
2007	F336	218	218
2007	F337	223	227
2007	F341	210	234
2007	F344	200	200
2007	F348	176	200
2007	F349	190	227
2007	F351	177	234
2007	F354	210	210
2007	F356	176	210

2007	F359	226	227
2007	F360	183	234
2007	F363	186	270
2007	F365	186	234
2007	F368	183	243
2007	F369	185	230
2007	F370	227	252
2007	F386	210	243
2007	F388	183	243
2007	F389	218	226
2007	F400	185	185
2007	F402	234	234
2007	F405	210	243
2007	F407	186	186
2007	F413	199	243
2007	F430	223	223
2007	F437	218	218
2007	F442	183	195
2007	F443	184	200
2007	F444	210	210
2007	F445	184	210
2007	F452	197	197
2007	F453	226	234
2007	F455	182	210
2007	F456	183	210
2007	F457	189	227
2007	F458	189	190
2007	F459	184	227
2007	F460	184	184
2007	F461	195	195
2007	F462	243	243
2007	F463	183	243
2007	F464	175	234
2007	F465	182	223
2007	F466	194	194
2007	F467	176	176
2007	F468	164	164
2007	F469	210	243
2007	F470	214	227
2007	F471	210	243
2007	F472	189	226
2007	F473	195	195
2007	F474	213	223
2007	F475	210	210
2008	F002	197	220
2008	F016	177	177
2008	F025	199	204
2008	F028	270	270
2008	F029	187	193

2008	F039	199	205
2008	F045	189	189
2008	F054	183	200
2008	F059	183	259
2008	F066	259	259
2008	F071	200	204
2008	F096	177	242
2008	F098	199	245
2008	F099	203	219
2008	F100	218	245
2008	F102	189	235
2008	F119	199	199
2008	F139	188	226
2008	F150	186	189
2008	F151	217	221
2008	F155	201	219
2008	F170	203	203
2008	F172	177	220
2008	F173	203	221
2008	F182	183	183
2008	F183	199	200
2008	F193	189	189
2008	F200	188	193
2008	F203	177	259
2008	F207	199	209
2008	F212	218	219
2008	F221	259	259
2008	F222	183	193
2008	F231	200	201
2008	F237	200	219
2008	F238	177	245
2008	F242	199	220
2008	F244	199	204
2008	F245	200	226
2008	F247	199	234
2008	F252	188	209
2008	F258	199	220
2008	F267	218	220
2008	F268	200	219
2008	F280	177	205
2008	F291	177	221
2008	F299	183	205
2008	F301	219	223
2008	F303	183	199
2008	F304	186	189
2008	F305	186	245
2008	F307	203	204
2008	F320	188	220
2008	F323	183	183

2008	F341	203	209
2008	F344	189	219
2008	F345	203	219
2008	F348	217	218
2008	F351	209	209
2008	F357	188	189
2008	F360	177	270
2008	F363	177	201
2008	F365	199	203
2008	F368	199	235
2008	F369	177	177
2008	F370	226	226
2008	F372	199	220
2008	F388	217	235
2008	F400	226	226
2008	F421	199	217
2008	F453	199	199
2008	F455	199	199
2008	F457	200	242
2008	F459	217	218
2008	F463	183	183
2008	F465	183	183
2008	F475	189	209
2008	F476	177	245
2008	F477	183	193
2008	F478	199	204
2008	F479	183	204
2008	F480	242	245
2008	F481	189	219
2008	F483	199	200
2008	F484	183	204
2008	F485	199	205
2008	F486	187	204
2008	F487	204	204
2008	F488	188	189
2008	F489	187	203
2008	F490	183	204
2008	F491	199	200
2008	F492	177	221
2008	F493	183	183
2008	F494	204	204
2008	F496	189	209
2008	F497	189	189
2008	F498	188	204
2008	F499	205	221
2008	F500	200	204
2008	F501	200	203
2008	F502	177	188
2008	F503	218	226

2008	F504	204	205
2008	F505	203	221
2008	F507	199	203
2008	F508	189	189
2008	F509	188	189
2008	F510	199	201
2008	F511	242	242
2008	F512	183	203
2009	F004	197	225
2009	F008	192	205
2009	F028	188	188
2009	F039	190	236
2009	F040	255	255
2009	F045	198	226
2009	F049	190	255
2009	F059	202	249
2009	F066	223	226
2009	F071	202	202
2009	F072	241	241
2009	F084	189	189
2009	F098	216	255
2009	F099	189	205
2009	F100	173	226
2009	F102	173	255
2009	F109	192	192
2009	F119	223	255
2009	F121	197	197
2009	F148	219	219
2009	F155	253	255
2009	F165	249	249
2009	F167	198	203
2009	F170	198	204
2009	F172	198	233
2009	F176	227	227
2009	F181	216	216
2009	F183	197	202
2009	F189	203	210
2009	F200	255	255
2009	F231	228	228
2009	F235	248	248
2009	F238	189	189
2009	F240	255	255
2009	F241	228	255
2009	F242	226	228
2009	F243	235	255
2009	F245	197	197
2009	F251	255	255
2009	F252	173	221
2009	F267	205	224

2009	F268	189	189
2009	F274	227	248
2009	F280	192	225
2009	F291	209	228
2009	F300	249	249
2009	F301	198	225
2009	F306	241	241
2009	F312	189	190
2009	F313	190	192
2009	F316	255	255
2009	F318	223	228
2009	F320	198	227
2009	F323	223	223
2009	F326	189	189
2009	F337	255	255
2009	F344	198	198
2009	F345	223	233
2009	F348	198	198
2009	F356	248	248
2009	F357	204	204
2009	F360	203	236
2009	F368	190	255
2009	F369	202	202
2009	F374	214	228
2009	F388	173	173
2009	F389	224	224
2009	F402	224	225
2009	F410	255	255
2009	F419	169	169
2009	F421	248	248
2009	F430	223	228
2009	F443	173	173
2009	F444	241	248
2009	F453	189	190
2009	F457	203	228
2009	F462	248	248
2009	F474	223	223
2009	F479	192	192
2009	F489	198	203
2009	F490	190	255
2009	F491	233	255
2009	F492	191	202
2009	F499	233	233
2009	F503	197	228
2009	F506	202	219
2009	F511	198	255
2009	F513	223	228
2009	F515	253	253
2009	F516	223	223

2009	F517	235	237
2009	F519	248	248
2009	F520	255	255
2009	F521	219	221
2009	F522	249	249
2009	F523	197	255
2009	F524	190	236
2009	F525	198	225
2009	F526	255	255
2009	F527	198	225
2010	F002	178	270
2010	F016	211	224
2010	F039	194	224
2010	F045	205	205
2010	F049	201	225
2010	F059	156	215
2010	F066	173	174
2010	F098	192	223
2010	F099	173	225
2010	F100	161	227
2010	F102	192	225
2010	F119	174	212
2010	F121	216	216
2010	F139	185	215
2010	F155	192	225
2010	F172	236	236
2010	F181	224	255
2010	F183	192	212
2010	F187	185	216
2010	F189	161	161
2010	F200	161	224
2010	F207	205	215
2010	F217	255	255
2010	F230	192	215
2010	F243	203	215
2010	F245	185	224
2010	F265	185	185
2010	F268	174	212
2010	F280	184	225
2010	F291	185	261
2010	F299	161	225
2010	F303	211	225
2010	F307	192	205
2010	F310	222	255
2010	F312	200	212
2010	F318	171	225
2010	F323	201	226
2010	F336	255	255
2010	F337	201	201

2010	F345	185	225
2010	F348	156	187
2010	F351	211	226
2010	F371	246	246
2010	F374	222	222
2010	F378	185	185
2010	F389	161	161
2010	F402	214	215
2010	F405	200	203
2010	F407	222	226
2010	F419	161	227
2010	F455	211	222
2010	F462	214	215
2010	F463	202	205
2010	F467	161	161
2010	F469	216	216
2010	F493	192	211
2010	F499	223	227
2010	F507	205	215
2010	F521	200	226
2010	F532	246	246
2010	F533	201	225
2010	F534	185	203
2010	F535	225	226
2010	F536	200	227
2010	F537	161	161
2010	F538	236	236
2010	F539	212	226
2010	F540	173	174
2010	F541	211	227
2010	F542	223	226
2010	F543	261	261
2010	F544	222	227
2010	F545	171	269
