# Supplementary material for: Adapting to a Warmer Ocean—Seasonal Shift of Baleen Whale Movements over Three Decades
Source: PLoS One. 2015 Mar 18;10(3):e0121374. doi: 10.1371/journal.pone.0121374 (PMC4364899; doi:10.1371/journal.pone.0121374)
Supplement: S1 Table — Mean arrival and departure date for fin and (mature) humpback whales and the effort covariates according to Table 1 (No of survey days in June, July, June and July, and the first survey day of the year) (DOCX) [file pone.0121374.s010.docx]

S1 Table: Dataset. Mean arrival and departure date for fin and (mature) humpback whales and the effort covariates according to Table1 (No of survey days in June, July, June and July, and the first survey day of the year)

|  | Fin whales | | | | Humpback whale | | |  | | | Effort data | |  | |  | |  |
| --- | --- | --- | --- | --- | --- | --- | --- | --- | --- | --- | --- | --- | --- | --- | --- | --- | --- |
| year | sample size | mean arrival | mean departure | sample size | | mean arrival | mean departure | | No days June | No days July | | No days June+July | | first day | |  |  |
| 1984 | 11 | 238.55 | 243.64 |  | |  |  | | 0 | 1 | | 1 | | 218 | |  |  |
| 1985 | 14 | 243.14 | 243.21 |  | |  |  | | 0 | 11 | | 11 | | 193 | |  |  |
| 1986 | 21 | 234.52 | 240.90 |  | |  |  | | 0 | 13 | | 13 | | 172 | |  |  |
| 1987 | 31 | 204.71 | 216.35 | 4 | | 267.50 | 274.25 | | 11 | 17 | | 28 | | 153 | |  |  |
| 1988 | 30 | 208.27 | 214.83 | 9 | | 245.11 | 280.38 | | 9 | 16 | | 25 | | 163 | |  |  |
| 1989 | 35 | 231.17 | 235.91 | 15 | | 254.13 | 288.60 | | 15 | 21 | | 36 | | 157 | |  |  |
| 1990 | 28 | 239.68 | 242.61 | 14 | | 249.28 | 277.21 | | 12 | 16 | | 28 | | 160 | |  |  |
| 1991 | 36 | 222.44 | 229.31 | 12 | | 241.25 | 249.50 | | 13 | 18 | | 31 | | 157 | |  |  |
| 1992 | 87 | 224.70 | 232.94 | 21 | | 237.76 | 278.77 | | 16 | 20 | | 36 | | 141 | |  |  |
| 1993 | 73 | 207.55 | 217.47 | 18 | | 212.94 | 240.00 | | 19 | 16 | | 35 | | 155 | |  |  |
| 1994 | 19 | 228.05 | 236.21 | 18 | | 233.00 | 236.44 | | 9 | 19 | | 28 | | 169 | |  |  |
| 1995 | 13 | 210.85 | 216.62 | 7 | | 216.71 | 217.29 | | 13 | 25 | | 38 | | 157 | |  |  |
| 1996 | 12 | 203.33 | 206.92 | 13 | | 226.38 | 229.62 | | 17 | 19 | | 36 | | 158 | |  |  |
| 1997 | 19 | 217.68 | 222.26 | 35 | | 236.42 | 254.66 | | 13 | 23 | | 36 | | 169 | |  |  |
| 1998 | 20 | 227.60 | 228.25 | 39 | | 244.64 | 263.95 | | 9 | 21 | | 30 | | 163 | |  |  |
| 1999 | 21 | 211.40 | 213.15 | 30 | | 224.10 | 259.07 | | 8 | 15 | | 23 | | 170 | |  |  |
| 2000 | 39 | 208.69 | 221.59 | 33 | | 225.51 | 255.64 | | 4 | 20 | | 24 | | 172 | |  |  |
| 2001 | 47 | 214.11 | 228.47 | 44 | | 230.47 | 263.20 | | 10 | 16 | | 26 | | 161 | |  |  |
| 2002 | 38 | 210.95 | 221.39 | 14 | | 223.14 | 235.71 | | 12 | 16 | | 28 | | 157 | |  |  |
| 2003 | 36 | 223.25 | 232.94 | 46 | | 231.89 | 255.93 | | 11 | 18 | | 29 | | 159 | |  |  |
| 2004 | 99 | 215.53 | 235.63 | 31 | | 224.83 | 254.87 | | 4 | 18 | | 22 | | 174 | |  |  |
| 2005 | 103 | 194.12 | 208.76 | 49 | | 223.14 | 251.51 | | 8 | 22 | | 30 | | 169 | |  |  |
| 2006 | 154 | 211.82 | 230.06 | 38 | | 218.73 | 234.03 | | 8 | 18 | | 26 | | 167 | |  |  |
| 2007 | 134 | 198.35 | 221.45 | 39 | | 206.02 | 227.51 | | 7 | 18 | | 25 | | 164 | |  |  |
| 2008 | 111 | 197.08 | 212.72 | 49 | | 222.38 | 255.82 | | 3 | 16 | | 19 | | 169 | |  |  |
| 2009 | 100 | 213.59 | 226.60 | 44 | | 219.91 | 243.59 | | 5 | 17 | | 22 | | 169 | |  |  |
| 2010 | 73 | 198.05 | 219.17 | 55 | | 223.62 | 243.44 | | 11 | 16 | | 27 | | 156 | |  |  |
