# Supplementary material for: Adapting to a Warmer Ocean—Seasonal Shift of Baleen Whale Movements over Three Decades
Source: PLoS One. 2015 Mar 18;10(3):e0121374. doi: 10.1371/journal.pone.0121374 (PMC4364899; doi:10.1371/journal.pone.0121374)
Supplement: S2 Table — Estimated regression slopes (SE) for the annual trend in arrival, using different subsets of the data and different model approaches. Numbers in bold italics indicate results used in the manuscript. In mixed effect models, individual whales were defined as random effects. (DOCX) [file pone.0121374.s011.docx]

**S2 Table. Model Results** Estimated regression slopes (SE) for the annual trend in arrival, using different subsets of the data and different model approaches. Numbers in bold italics indicate results used in the manuscript. In mixed effect models, individual whales were defined as random effects.

|  | Fin whale data set | Humpback whale data set |
| --- | --- | --- |
| Linear model (all data) | ***-1.062 (0.093)*** | -0.938 (0.102) |
| Linear model (animals seen more often than one day a season) | -0.749 (0.118) | -0.734 (0.114) |
| Linear model (mature only) |  | -***1.201 (0.135)*** |
| Mixed effect model (fin whale all data) humpback whales (mature only) | -1.115 (0.096) | -1.232 (0.138) |
